# Metal–organic framework composites with luminescent gold(iii) complexes. Strongly emissive and long-lived excited states in open air and photo-catalysis†
†Electronic supplementary information (ESI) available: Synthesis and characterization data, photo-catalytic studies, and supplementary figures/tables. X-ray crystallographic data of MOF1. CCDC 993259. For ESI and crystallographic data in CIF or other electronic format see DOI: 10.1039/c5sc02216a


**DOI:** 10.1039/c5sc02216a

**Published:** 2015-09-21

**Authors:** Chun-Yi Sun, Wai-Pong To, Xin-Long Wang, Kaai-Tung Chan, Zhong-Min Su, Chi-Ming Che

**Affiliations:** a State Key Laboratory of Synthetic Chemistry , Institute of Molecular Functional Materials , HKU-CAS Joint Laboratory on New Materials , Department of Chemistry , The University of Hong Kong , Pokfulam Road , Hong Kong , China . Email: cmche@hku.hk; b Institute of Functional Materials Chemistry , Northeast Normal University , Changchun , Jilin 130024 , China; c HKU Shenzhen Institute of Research and Innovation , Shenzhen , 518053 , China

## Abstract

The encapsulation of luminescent gold(iii) complexes by metal–organic frameworks (MOFs) lays the groundwork for new phosphorescent materials with activities that are not readily achieved by the host MOF materials or gold(iii) complexes alone. In this work, strong phosphorescence with lifetimes of up to ∼50 μs in open air at room temperature has been achieved by incorporation of cationic cyclometalated gold(iii) complexes into MOFs with anionic frameworks to form Au^III^@MOFs. The Au^III^@MOFs display solid state two-photon-induced phosphorescence. Photo-reduction of methyl viologen to the reduced radical was achieved inside Au^III^@MOFs and in the presence of Et_3_N upon excitation at *λ* > 370 nm under ambient conditions. These Au^III^@MOFs comprise a class of reusable and size-selective heterogeneous photo-catalysts for the aerobic oxidation of secondary amines to imines as well as five other reactions, including oxidative C–H functionalization under aerobic conditions.

## Abstract 2

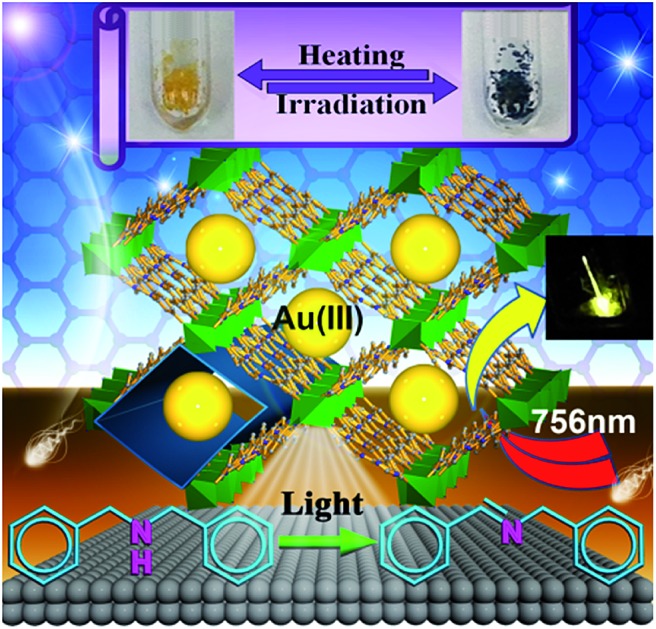
Encapsulation of luminescent gold(iii) complexes by metal–organic frameworks results in enhanced phosphorescence in open air, solid state two-photon-induced phosphorescence, and reusable photo-catalysts under aerobic conditions.

## Introduction

Metal–organic frameworks (MOFs) have received burgeoning interest over the past decades, with applications in gas storage/separation, proton/electrical conductivity, biomedicines, sensing, and catalysis.^1^,^2^ The tuning of both the metal nodes and the organic linkers in MOFs, by either pre- or post-synthetic^1^,^3^ methods involving covalent/dative bond formation, leads to extraordinary versatility in framework structure, porosity, and function. Encapsulation of a functional cargo into the pores of MOFs *via* non-covalent and/or dative interactions,^4^ such as the use of MOFs to encapsulate metal catalysts or luminescent metal ions/complexes by the cation exchange method,4*a* is an alternative approach to MOF modification. Pores with defined environments inside MOFs constitute a unique platform to confine guest species, and as a result, new functions/performance of the encapsulated guest species may emerge.

**Scheme 1:**
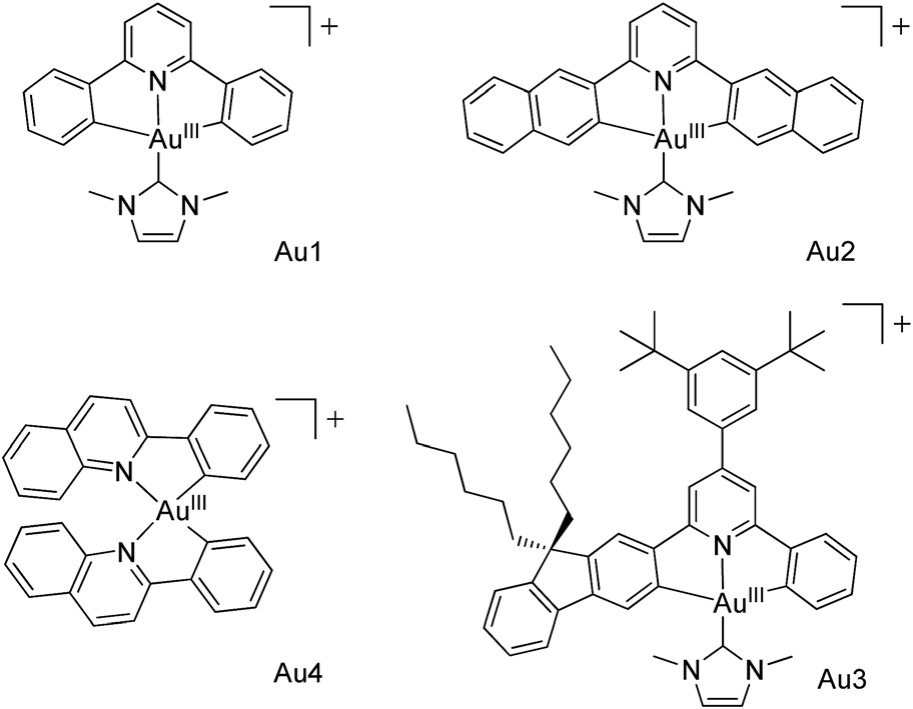
Au^III^ complexes Au1–Au4 used in this work.

We are attracted to using MOFs for housing metal complexes with long-lived emissive excited states for heterogeneous photochemical catalysis and luminescence applications. In the literature, other porous materials besides MOFs, such as mesoporous silica, have also been employed to encapsulate photoluminescent and/or photochemically active compounds,^5^,^6^ including confinement of a photoluminescent trinuclear gold(i) pyrazolate complex in the channels of mesoporous silica.6*b* Our attention has been directed towards recently reported luminescent gold(iii) complexes which display strongly emissive excited states with long lifetimes of up to 506 μs (emission quantum yields: up to 11.4%) in degassed solutions at room temperature.^7^ These complexes may have useful applications in photo-catalysis and emission sensing, particularly if they are located in an aerobic environment in which their photo-physical characteristics can be retained, and if the photochemical reactions they catalyse can be endowed with specificity. Here we report our findings on the encapsulation of strongly luminescent gold(iii) complexes with various dimensions (Au1–Au4,^7^,^8^ Scheme 1; Table S1 in the ESI†) by two MOFs with differing porous structures. The encapsulation was performed using a cation exchange method.4a,^9^ The Au^III^–MOF composites (Au^III^@MOFs) display long emission lifetimes of up to 48.8 μs at room temperature and in open air, exhibit solid state two-photon-induced phosphorescence, and can act as selective and recyclable catalysts for light-induced electron transfer reactions and aerobic C–N/C–C/C–O bond forming reactions. Of note, Corma and co-workers^10^ have described a recyclable Au^III^-functionalized MOF catalyst for domino coupling/cyclization and alkene hydrogenation, prepared by post-synthetic dative bond formation.

## Results and discussion

### Syntheses and structures of MOF1 and MOF2

Two MOFs, MOF1 and MOF2, built from similar ligands but possessing different types of pores with suitable window sizes, were chosen in this work to allow investigation of the effect of porous structure on the luminescence of Au(iii) complexes.

MOF1, formulated as [Me_2_NH_2_]_2_[Cd_2_(TATMB)_2_]·10DMF·3H_2_O (H_3_TATMB = 3,3′,3′′-((1,3,5-triazine-2,4,6-triyl)tris(azanediyl))tribenzoic acid) based on single-crystal X-ray diffraction, elemental analysis, thermogravimetric analysis (TGA), and charge-balance consideration, was obtained as colourless rod-like crystals by the reaction of H_3_TATMB with Cd(NO_3_)_2_·6H_2_O in DMF at 120 °C for 2 days (the [Me_2_NH_2_]^+^ cations came from decarbonylation of DMF^11^). The phase purity of MOF1 was examined by powder X-ray diffraction (PXRD) analysis.

**Fig. 1:**
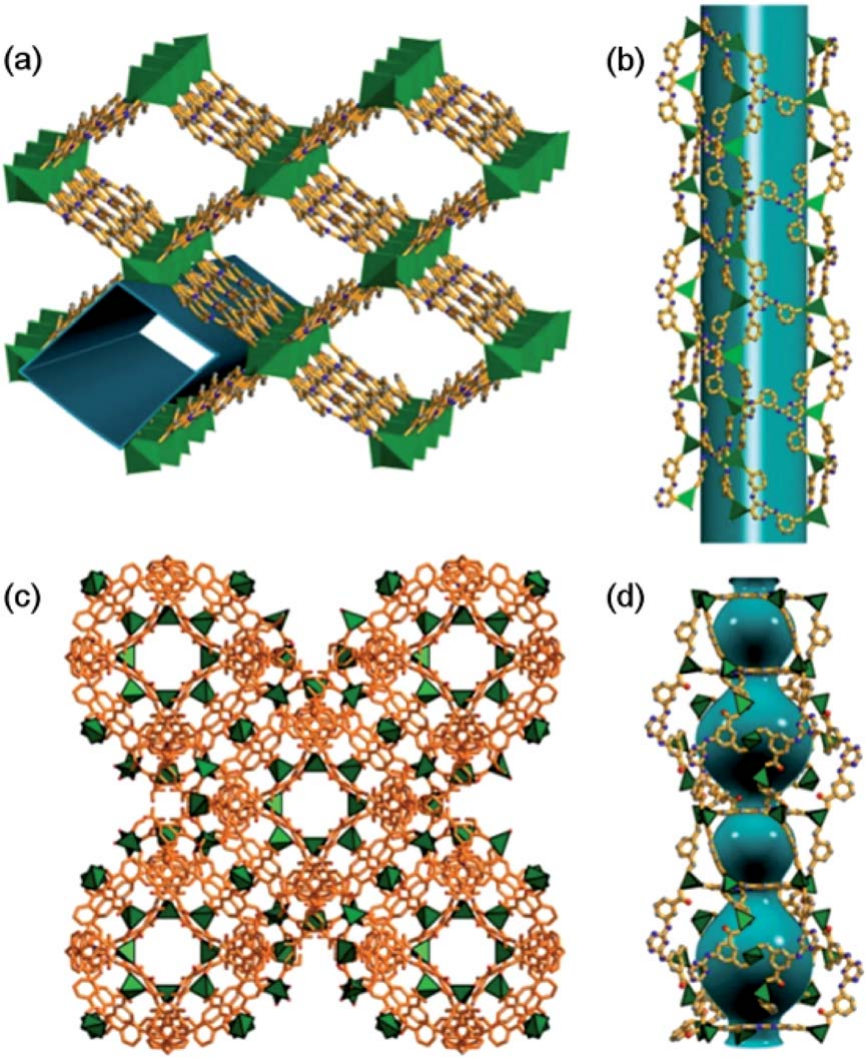
(a) 3D polyhedral structure of MOF1, viewed along the *c* axis; (b) 1D mesoporous nanotube component of MOF1; (c) 3D polyhedral structure of MOF2, viewed along the *a* axis; (d) trigonal and hexagonal prism-shaped nanocages in MOF2.

Single-crystal X-ray structure determination of MOF1 revealed a 3D framework (Fig. 1a) that can be described as a 4-connected uninodal net with hxg-d-4-C2/m topology^12^ (Fig. S1d, ESI†). The asymmetric unit contains one crystallographically independent Cd^2+^ ion and one TATMB ligand (the other cations and guest molecules are disordered and have not been precisely defined). The Cd^2+^ ion is seven-coordinated by six carboxylate oxygen atoms of three TATMB ligands and one N atom from the triazine ring of another TATMB ligand; each pair of nearest-neighbour Cd^2+^ ions are bridged by two TATMB ligands to form an 18-membered ring (Fig. S1a, ESI†). There are large nano-scale tubular channels (window size: 17 × 24 Å^2^, Fig. 1b) running along the [023] plane, with solvent molecules and [Me_2_NH_2_]^+^ ions residing in the channels. The effective free volume of MOF1 is 70.7% of the crystal volume (calculated by PLATON analysis^13^). The TGA data (Fig. S2, ESI†) revealed a decrease in weight of 38.4%, attributed to a loss of solvent and water molecules; the de-solvated framework remained stable until ∼290 °C (above this temperature the framework began to decompose and the final residue was CdO [6.32% observed, 6.20% calculated]). N_2_ adsorption measurements for MOF1 revealed an isotherm (Fig. S3, ESI†) which corresponds to a Type 1 adsorption isotherm for porous materials.^14^

MOF2, [Me_2_NH_2_]_2_[Zn(TATAT)_2/3_]·3DMF·H_2_O (TATAT = 5,5′,5′′-(1,3,5-triazine-2,4,6-triyl)tris(azanediyl)triisophthalate), was prepared according to the literature.^15^ MOF2 has a 3D meso-cage structure (Fig. 1c) featuring alternately arranged hexagonal prismatic cages (window size: 6.3 × 10.5 Å^2^) and trigonal prismatic cages (window size: 14.3 × 11.5 Å^2^) (Fig. 1d), with a potential solvent volume that accounts for 60% of the empty volume.^15^ The [Me_2_NH_2_]^+^ cations and DMF/H_2_O molecules reside in these nano-scale cages.

### Syntheses, characterization and photo-physical properties of Au^III^@MOF composites

**Fig. 2:**
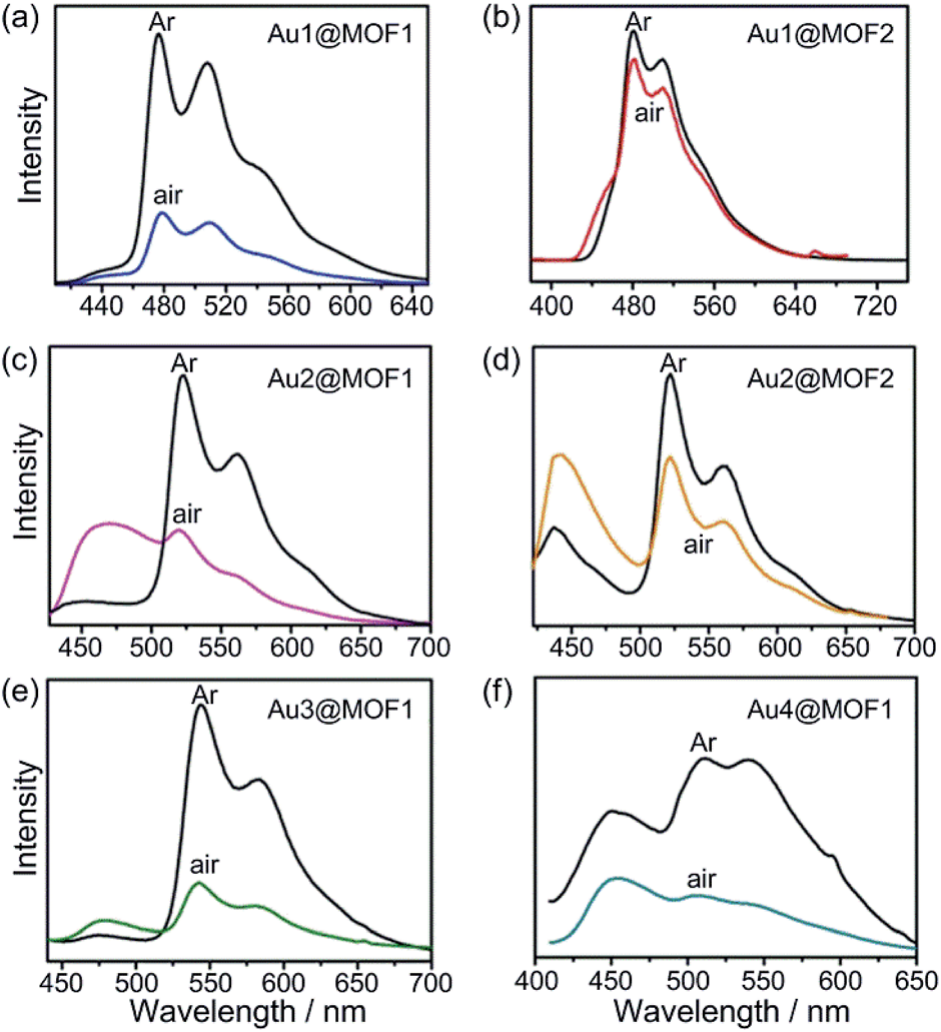
Emission spectra of Au^III^@MOFs in Ar (black lines) and air (color lines) upon excitation at *λ* = 365 nm at room temperature. (a) Au1@MOF1; (b) Au1@MOF2; (c) Au2@MOF1; (d) Au2@MOF2; (e) Au3@MOF1; (f) Au4@MOF1.

Au^III^@MOFs, including Au^III^@MOF1 (Au^III^ = Au1–Au4) and Au^III^@MOF2 (Au^III^ = Au1, Au2), were obtained as yellow or pale yellow solids (Fig. S4, ESI†) by immersing MOF1 or MOF2 in a DMF solution of the CF_3_SO_3_^–^ or BF_4_^–^ salt of the corresponding Au^III^ complex for several days. The incorporated Au^III^ complexes, comprising 0.91–8.26 wt% of the Au^III^@MOFs (determined by inductively coupled plasma (ICP) spectroscopy, Table S2, ESI†), are likely to reside in the inner pores of the MOF host materials based on the following: (i) the MOF hosts of these Au^III^@MOFs contain nanochannels/nanocages with window sizes larger than the sizes of the incorporated Au^III^ complexes (Table S1, ESI†); (ii) complex Au3, which is larger than the window sizes of the nano-cages in MOF2, did not give Au3@MOF2 (under similar conditions the MOF2 in the reaction mixture remained colourless, and ICP analysis revealed no Au3 incorporation into MOF2), suggesting that adsorption of the Au^III^ complex outside the pores (or in gaps/cracks between crystal grains) of the MOF is negligible; (iii) the PXRD data of the Au^III^@MOFs were nearly identical to those of the corresponding MOF matrices (Fig. S5, ESI†); (iv) examination of the cross section of a crystal of Au2@MOF2 (1.43 wt% Au2) by optical microscopy under UV light irradiation at 365 nm showed the characteristic yellowish green emission of Au2 (ref. 7), congruent with incorporation of Au2 inside the crystal (Fig. S8, ESI†); (v) further analysis of the cross section of Au2@MOF2 by scanning electron microscopy (SEM) and electron dispersive X-ray spectroscopy (EDX) revealed that Au2 was disorderedly dispersed at the cross section (Fig. S9, ESI†) and that no F was detected in Au2@MOF2 by EDX, even when the content of incorporated Au2 increased from 1.43 to 8.26 wt% (Fig. S10, ESI†); (vi) N_2_ adsorption experiments (Fig. S11, ESI†) showed that, from free MOF2 to Au2@MOF2 (1.43 wt% Au2), the pore volume decreased from 0.303 to 0.276 cm^3^ g^–1^, with a decrease in BET surface area from 1112 to 1011 m^2^ g^–1^. Increasing the Au2 content of Au2@MOF2 to 8.26 wt% led to a reduction in the pore volume and BET surface area to 0.183 cm^3^ g^–1^ and 656 m^2^ g^–1^, respectively. The decreases in the pore volume and BET surface area are attributed to the encapsulation of Au2 in the pores of MOF2. Au^III^@MOFs display strong emission under air at room temperature. As depicted in their emission spectra (Fig. 2), two emission bands are observed. The one at ∼450 nm corresponds to the MOF host. The other is a vibronic structured emission with peak *λ*_max_ at 478–550, 520–610, 540–630 and 510–580 nm for Au^III^ = Au1, Au2, Au3 and Au4, respectively. These bands are all similar to those observed in the emission spectra of the corresponding Au^III^ complexes in degassed solution.^7^,^8^ The measured emission lifetimes (*τ*_0_), quantum yields (*φ*_em_), and estimated radiative (*k*_r_) and non-radiative (*k*_nr_) decay rate constants of Au^III^@MOFs and Au1–Au4 under different conditions are compiled in Table 1.

**Table 1:** Emission lifetimes, quantum yields, and radiative and non-radiative decay rate constants of the Au^III^ complexes and Au^III^@MOFs

Compound	Conditions	*λ*_max_ (nm)	*τ*_0_ (μs)	*φ*_em_ (%)	*k*_r_ ^*a*^ (10^3^ s^–1^)	*k*_nr_ ^*b*^ (10^5^ s^–1^)
Au1 solid	Air	478, 511	0.20			
Au1 (DMF solution)	Air	478, 511	0.32	0.06	1.88	31.23
	Degassed	478, 511	0.59	0.09	1.53	16.93
Au1@MOF1	Air	478, 511	10.1	1.3	1.29	0.98
	Ar	478, 511	33.6	3.5	1.04	0.29
Au1@MOF2	Air	478, 511	19.4	2.0	1.03	0.51
	Ar	478, 511	21.2	2.7	1.27	0.46
Au2 (DMF solution)	Air	Non-emissive				
	Degassed	520, 558	21.4	0.64	0.30	0.46
Au2@MOF1	Air	520, 558	7.9	1.1	1.39	1.25
	Ar	520, 558	146.3	11.4	0.78	0.06
Au2@MOF2^*c*^	Air	520, 558	14.5	1.5	1.03	0.68
	Ar	520, 558	98.6	8.1	0.82	0.09
	O_2_^*d*^	520, 558	6.0			
Au2@MOF2^*e*^	Air	522, 560	15.8			
	Ar	522, 560	104.2			
Au3 (DMF solution)	Air	540, 580	0.88	0.09	1.02	11.35
	Degassed	540, 580	117.7	7.0	0.60	0.08
Au3@MOF1	Air	540, 580	48.8	11.9	2.44	0.18
	Ar	540, 580	130.7	34.8	2.66	0.05
Au4 (DMF solution)	Air	510, 540	0.52	0.12	2.31	19.21
	Degassed	510, 540	8.0	0.95	1.19	1.24
Au4@MOF1	Air	510, 540	0.97	0.20	2.06	10.29
	Ar	510, 540	21.7	3.9	1.80	0.44

^*a*^Radiative decay rate constant estimated by *k*_r_ = *φ*/*τ*.

^*b*^Non-radiative decay rate constant estimated by *k*_nr_ = (1 – *φ*)/*τ*.

^*c*^Au2 content: 1.43 wt%.

^*d*^Au2@MOF2 immersed in acetonitrile saturated with O_2_.

^*e*^Au2 content: 8.26 wt%.

Encapsulation of Au1–Au4 by MOF1 or MOF2 was observed to cause significant increases in the intensity and lifetime of the emission of these Au^III^ complexes (Fig. 2 and Table 1; Fig. S6 and S7, ESI†). As an example, at room temperature, Au2 is non-emissive in the solid state, and the intensity of its emission at 520 nm in degassed dichloromethane solution decreased more than 100-fold upon exposure of the solution to air.^7^ Remarkably, Au2@MOF2 is luminescent under aerobic conditions,^16^ and the quantum yield decreased only ∼5-fold compared with that measured under argon (Table 1). For Au2@MOF1 and Au4@MOF1, the decrease in emission quantum yield on changing the atmosphere from argon to air is ∼10- and ∼20-fold, respectively. Moreover, while Au2 is virtually non-emissive in DMF solution in air, Au2@MOF2 (1.43 wt% Au2), Au2@MOF2 (8.26 wt% Au2), and Au2@MOF1 are luminescent in air, with emission lifetimes of 14.5, 15.8, and 7.9 μs, respectively. Notably, the emission lifetime of Au1@MOF2 in air is 19.4 μs, which is >60-fold longer than that of Au1 in the solid state (0.20 μs) and in DMF solution (0.32 μs) under aerobic conditions (Table 1).^17^ The emission lifetime of Au3@MOF1 in open air is 48.8 μs, the longest emission lifetime reported for luminescent Au^III^ complexes under aerobic conditions. Compared with Au^III^@MOF2, the Au^III^@MOF1 composites are more susceptible to luminescence quenching under aerobic conditions (Fig. 2 and Table 1), due probably to the larger window size of the pores in MOF1 than in MOF2. The intrinsic triplet excited state properties and molecular sizes of the Au^III^ complexes could also affect the luminescence quenching behaviour of Au^III^@MOFs upon exposure to air. The elevated intensities and lifetimes of the emissions of Au^III^@MOFs in the solid state under aerobic conditions are attributed to the following: (i) the emission lifetimes of Au^III^@MOFs under argon are longer, and their non-radiative decay rate constants (*k*_nr_) are smaller, than those of the corresponding Au^III^ complexes in degassed solution (Table 1). Electrostatic binding of the Au^III^ complexes on the surfaces of the inner pores of the MOFs would restrict the molecular motion of the Au^III^ complexes, thereby slowing down non-radiative decay and self-quenching (by diffusion) of the emissive excited state.^18^ The radiative decay rate constants (*k*_r_) of Au^III^@MOFs and Au1–Au4 are in the order of 10^2^ to 10^3^ s^–1^ (Table 1), typical of ^3^IL excited states of luminescent gold(iii) complexes.8*a* (ii) The steady state concentration of oxygen in the pores of Au^III^@MOFs may be lower than that of the oxygen dissolved in the solvent and in free atmosphere. This may reduce oxygen quenching of the emission of Au^III^@MOFs in air.^19^ Given that the activity of O_2_ in the matrix would equal that in the external environment if the matrix and the ambient environment are at equilibrium, and considering the use of O_2_ as a terminal oxidant in photo-catalytic reactions within the matrix (see the section below on the photo-catalytic properties of the Au^III^@MOF composites) as well as the partial luminescence quenching under aerobic conditions, O_2_ diffusion in the matrix of Au^III^@MOFs is likely to occur at a finite rate, with the mass transport of O_2_ through the matrix being sufficiently slow that the above-mentioned equilibrium did not fully occur.

### Light-induced electron transfer reactivity of Au2@MOF2

**Fig. 3:**
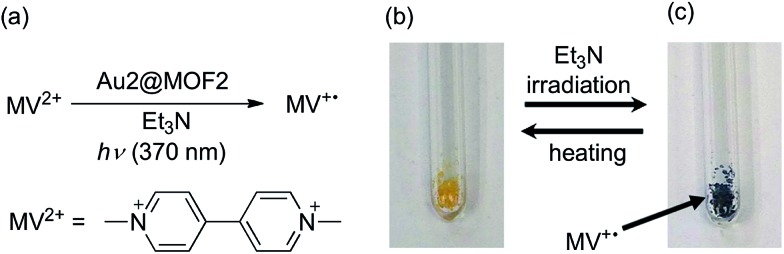
(a) Photochemical reaction (light source: xenon lamp) between Au2@MOF2 and MV^2+^, and photographs of the reaction mixture before (b) and after (c) irradiation with light for 1 min.

Au^III^@MOFs were observed to display light-induced electron transfer reactivity. As an example, Au2@MOF2 (1.43 wt% Au2) was first immersed into a solution of methyl viologen dication (MV^2+^) in DMF at room temperature for 12 h to allow encapsulation of MV^2+^ inside the pores of the MOF composite, followed by removal of the solution, washing with DMF and drying. Upon xenon lamp (*λ* > 370 nm) irradiation of a mixture of the resulting Au^III^@MOF–MV^2+^ and a drop of Et_3_N liquid in air, the characteristic blue colour of the MV^+^˙ radical appeared within a few seconds (the colour intensity increased with irradiation time), as depicted in Fig. 3. Heating the mixture (after removing the xenon lamp) to remove Et_3_N was observed to revert MV^+^˙ back to MV^2+^, and the photolysis was recycled three times. For comparison, irradiation of a mixture of Au2, MV^2+^ and Et_3_N in MeCN under the same aerobic conditions did not give a similar net change in colour of the solution mixture. In fact, a recently reported photochemical reaction of a Au^III^ complex with MV^2+^ was conducted under degassed conditions.8*a* We measured the N_2_ adsorption of Au2@MOF2–MV^2+^ without sample pretreatment, and found that very little N_2_ could be adsorbed by the sample (∼3.7 cm^3^ g^–1^). The corresponding BET surface area is 6.3 m^2^ g^–1^ (Fig. S12, ESI†), which is quite small compared with the BET surface area of the pretreated Au2@MOF2 (1011 m^2^ g^–1^). Thus, for Au2@MOF2, the observed photochemical reaction with MV^2+^ and Et_3_N in air can be attributed to the occurrence of the reaction inside the pores of the MOF composite, which could be occupied by solvent and Et_3_N molecules, thus decreasing the quenching of MV^+^˙ by O_2_.

### Two-photon absorption of Au3@MOF1 in the solid state

**Fig. 4:**
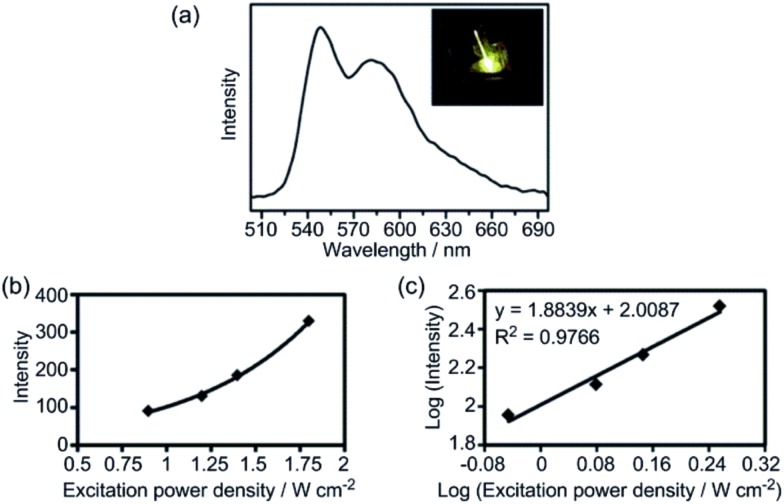
(a) Emission spectrum of Au3@MOF1 excited with 756 nm laser light (inset: photo of the emission of the solid); (b) plot of emission intensity against excitation power density (W cm^–2^); (c) double-log plot of emission intensity against excitation power density.

Two-photon absorption has useful applications in chemistry and biological science;^20^,^21^ the two-photon-excited emission of a phosphorescent Au^III^ complex in solution has recently been reported.8*a* However, there are few reported examples of this property in the solid state, owing to aggregation-induced quenching.^21^ In the present study, we observed that Au^III^@MOFs display two-photon-induced phosphorescence^22^ in the solid state. For Au3@MOF1, excitation at 756 nm with a focused laser beam gave bright yellow emission (Fig. 4a) with peak maxima at 545 and 585 nm (characteristic of the emission of Au3) and an intensity showing a quadratic dependence (*y* = *x*^2^) on the laser power density (Fig. 4b and c).

### Photo-catalytic properties of Au^III^@MOF composites

**Fig. 5:**
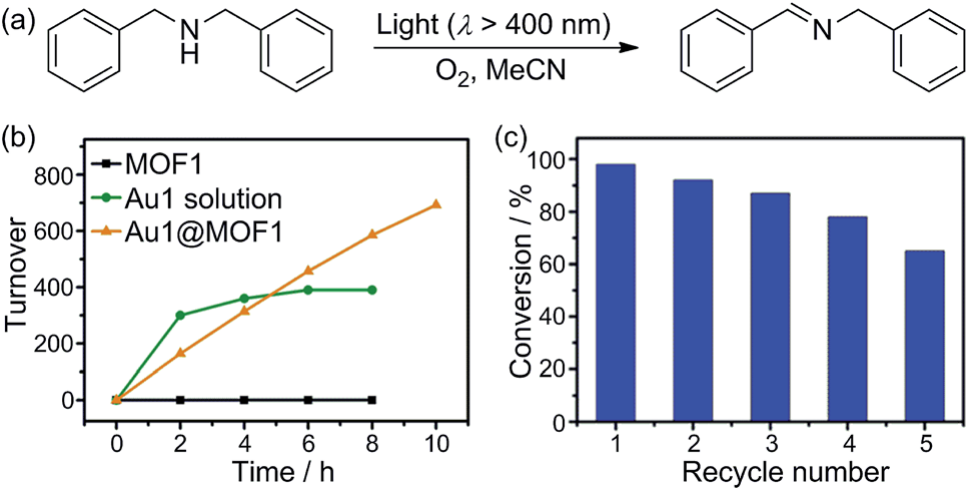
(a) Photo-catalytic oxidation of dibenzylamine to imine; (b) time course of the oxidation by Au1, MOF1, and Au1@MOF1; (c) substrate conversion in recycling experiments using the Au1@MOF1 catalyst.

**Scheme 2:**
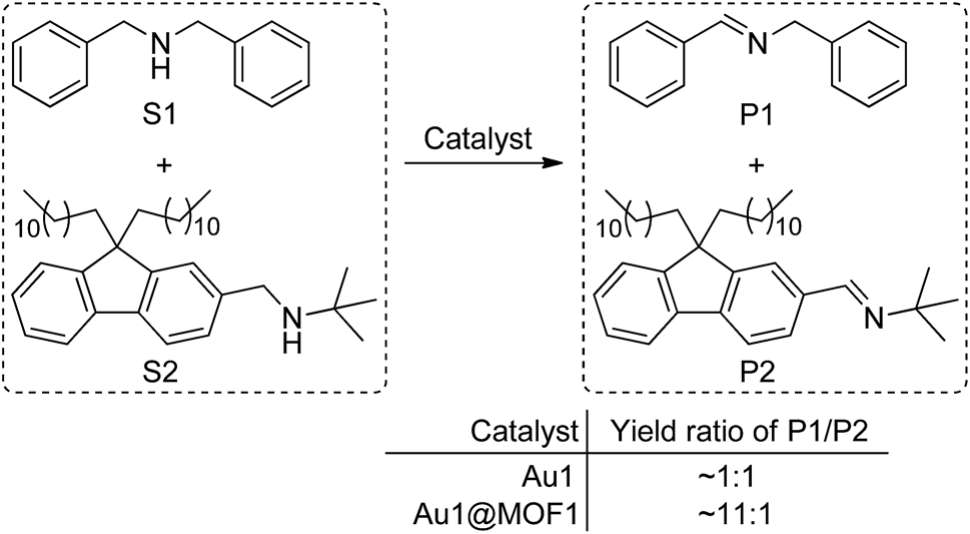
Competitive photo-catalytic oxidation of secondary amines.

Cyclometalated Au^III^ complexes have recently been reported to sensitize the formation of ^1^O_2_ upon light excitation for the oxidation of secondary amines to imines and for oxidative cyanation of tertiary amines.^7^,8a,^23^ Using the former as a paradigm, we examined the photo-catalytic activities of Au^III^@MOFs. The photochemical reactions were performed in MeCN. The solution mixture was under light irradiation (*λ* > 400 nm) with constant bubbling of oxygen. Control experiments showed that both MOF1 and MOF2 are stable in MeCN for at least 24 h, as revealed by PXRD measurements (see Fig. S13 of ESI†). The following was observed: (i) for the photo-reaction with dibenzylamine (Fig. 5), Au1@MOF1 gave the imine product with a turnover number of 692, exceeding the turnover number of 390 obtained with free Au1. The photo-activity of Au1@MOF1 showed little variation over a period of 10 h, whereas free Au1 exhibited a significant decrease in photo-activity after 2 h, with almost no imine product obtained after 6 h (Fig. 5b). (ii) The Au1@MOF1 catalyst could be recycled by washing with MeCN. After five cycles, the substrate conversion retained a value of ∼70% (Fig. 5c). (iii) The photo-catalytic activity is attributed to Au1 encapsulated by MOF1 rather than Au1 leached into solution. This is confirmed by the finding that the solution phase of the Au1@MOF1-catalysed reaction mixture remained colourless during the course of photolysis. This is in contrast to the reaction catalysed by free Au1, in which there was a colour change attributed to the decomposition of the Au1 catalyst after 6 h of photolysis. ICP measurements revealed nearly the same content of gold in the Au1@MOF1 sample before and after photo-catalysis; control experiments with MOF1 (free of Au) as the catalyst gave few product turnovers (Fig. 5b). (iv) Substrate size selectivity1b,2d was observed for the Au1@MOF1-catalysed competitive photochemical oxidation of dibenzylamine (S1) in the presence of another secondary amine of larger size (S2, Scheme 2). The photochemical reaction consumed S1 rapidly without significant consumption of S2, and the imine products P1 and P2 were formed in a yield ratio of ∼11 : 1 for a reaction time of 1.5 h. Under the same conditions, catalyst Au1 showed no selectivity in solution, with a P1/P2 yield ratio of ∼1 : 1 (Scheme 2). The drastic difference between the yields of P1 and P2 in the Au1@MOF1-catalysed reaction can be attributed to the reaction mainly occurring inside the channels of the MOF framework. The smaller substrate, S1, could enter the channels of MOF1, but the larger one, S2, could not; the formation of a small amount of P2 in the reaction mixture is attributed to the external diffusion of singlet oxygen (produced inside the MOF) and its subsequent reaction with S2. Au2@MOF1 and Au3@MOF1 were also found to catalyse the light-induced aerobic oxidation of dibenzylamine (Fig. S14 and S15, ESI†). The total turnover numbers of the imine product furnished by Au2@MOF1 (0.91 wt% Au2) and Au3@MOF1 (2.34 wt% Au3) were 557 and 920, respectively, and both were higher than the values of 510 and 760 obtained with catalysts Au2 and Au3, respectively. The Au2@MOF2 (1.43 wt% Au2) catalyst gave the imine product with turnover number of 610; changing the catalyst to Au2@MOF2 (8.26 wt% Au2) led to a ∼2-fold increase in conversion rate, albeit with a decrease in product turnover number (calculated based on % wt of Au2 in Au2@MOF2; Fig. S16, ESI†).

**Fig. 6:**
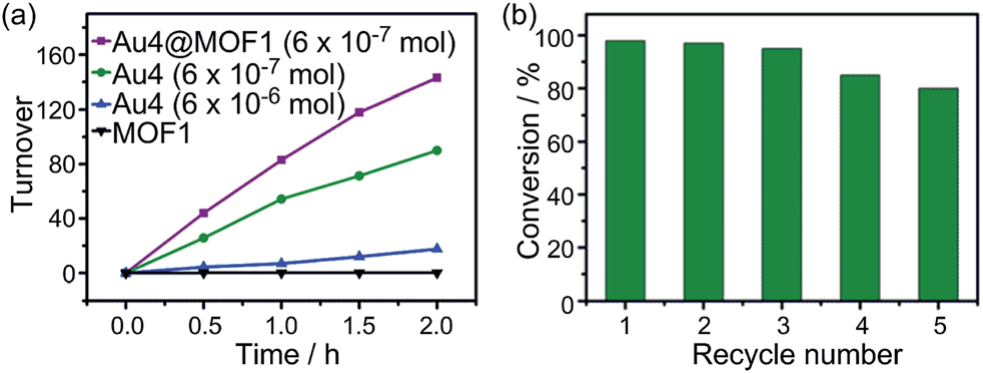
(a) Product turnovers in the photo-catalytic oxidation of dibenzylamine to imine by MOF1, Au4@MOF1, and Au4 solution; (b) substrate conversion in recycling experiments using the Au4@MOF1 catalyst.

The photochemical oxidation of dibenzylamine was also catalysed by Au4 and Au4@MOF1 (Au4: 6 × 10^–7^ mol in both cases). Au4@MOF1 was a more active catalyst with a turnover number of >143 attained within 2 h (Fig. 6a). Notably, the initial reaction rate of the Au4@MOF1 system was two-fold higher than that of Au4 alone. After recycling five times, the photo-activity of the Au4@MOF1 catalyst maintained ∼80% of its initial value (Fig. 6b). Increasing the content of Au4 in solution from 6 × 10^–7^ to 6 × 10^–6^ mol decreased the product turnover number from 90 to 18 (Fig. 6a).

**Scheme 3:**
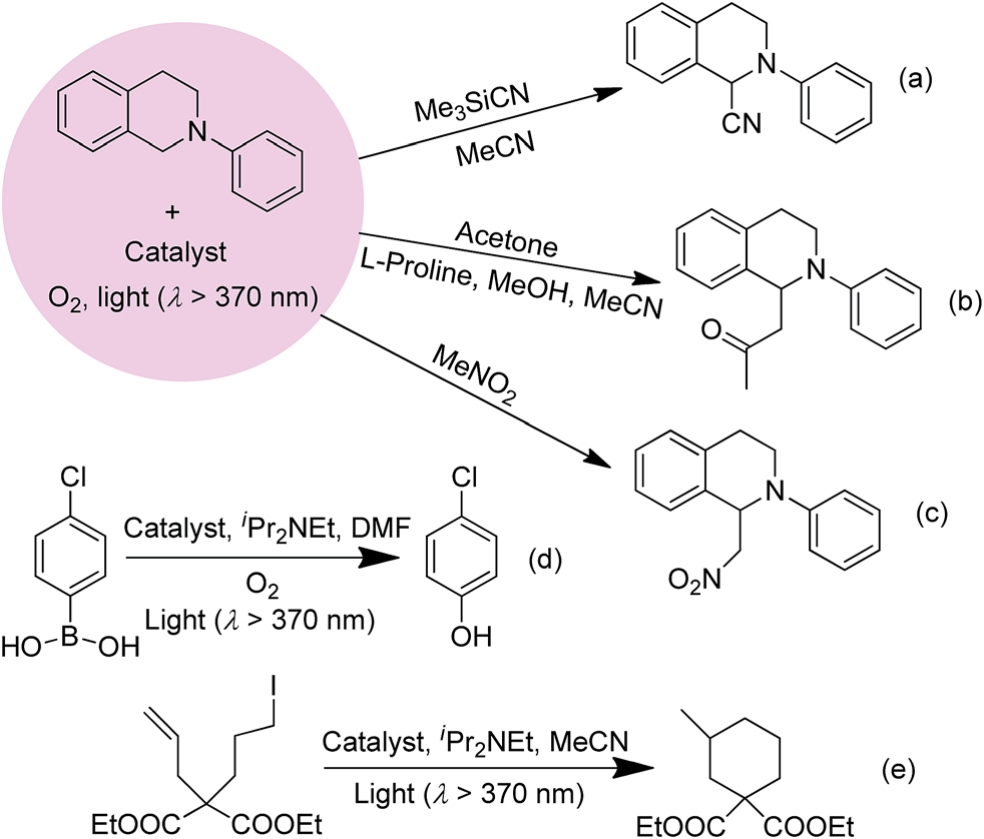
Photo-catalytic oxidative cyanation of a tertiary amine (a), Mannich-type reaction (b), aza-Henry reaction (c), hydroxylation of 4-chlorophenylboronic acid (d), and reductive cyclization of alkyl iodide (e) catalysed by Au^III^@MOFs.

The improvement in photo-catalytic activity of Au^III^@MOFs relative to the free Au^III^ complexes could be extended to the oxidative cyanation of a tertiary amine, a Mannich-type reaction, the aza-Henry reaction, the hydroxylation of 4-chlorophenylboronic acid, and the reductive cyclization of alkyl iodide.^24^ These findings are depicted in Scheme 3 and in Fig. S17 and Tables S3–5 of the ESI.† For example, the steady formation of product was observed over 8 h in the Au1@MOF1-catalysed oxidative cyanation of *N*-phenyl-1,2,3,4-tetrahydroisoquinoline, in contrast to a significant decrease in the activity of the free Au1 catalyst after 2.5 h of photolysis (Fig. S17, ESI†). The product yield obtained with the Au1@MOF1 catalyst was ∼81% (turnover number of 923). For the other photochemical reactions with Au2@MOF2 (1.43 wt% Au2) as catalyst, the products were obtained in 63–86% yields with turnover numbers of 137–549. These values were higher than the corresponding product yields and turnover numbers obtained with Au2 alone (Tables S3–S5, ESI†).

To gain further insight into the photochemical oxidation reactions catalysed by Au^III^@MOFs under aerobic conditions, which are likely to involve the formation of ^1^O_2_,^7^,23b we measured the reduction potentials, *E*(Au^0/–^), of Au1–Au4 (Table S6, ESI†) and estimated the excited state reduction potentials, *E*(Au*/Au^–^), from the electrochemical and emission data. The estimated *E*(Au*/Au^–^) values of Au1–Au4 range from +0.74 to +1.38 V *vs.* Cp_2_Fe^+/0^ (Table S6, ESI†), indicating that they are strong oxidants in the excited state. The potentials of dibenzylamine and *N*-phenyl-1,2,3,4-tetrahydroisoquinoline, *E*(amine^+/0^), were measured to be +0.50 V and +0.25 V *vs.* Cp_2_Fe^+/0^, respectively. Thus, the thermodynamic driving force for the reaction of the excited Au1–Au4 with dibenzylamine was estimated to be in the range of +0.24 to +0.88 V, whereas that of the excited Au2 complex with *N*-phenyl-1,2,3,4-tetrahydroisoquinoline is +0.49 V. Nanosecond time-resolved transient absorption (TA) measurements (Table S7, ESI†) revealed that, in the presence of dibenzylamine (0.1 M) in degassed DMF, the TA of Au2 and Au3 was quickly quenched (*τ*_TA_: 1.4 μs for Au2, 2.2 μs for Au3) and returned to the baseline, whereas the TA of Au1 and Au4 evolved to give long-lived species (*τ*_TA_: 145 μs for Au1, 24 μs for Au4), attributable to the formation of Au1^–^ and Au4^–^.^25^ The TA spectrum of Au2 in the presence of *N*-phenyl-1,2,3,4-tetrahydroisoquinoline (0.01 M) shows the formation of a long-lived species (*τ* = 65 μs) assignable to Au2^–^ (Table S8, ESI†). Thus, in the photochemical oxidation of dibenzylamine catalysed by Au1 and Au4, and that of *N*-phenyl-1,2,3,4-tetrahydroisoquinoline catalysed by Au2, the excited gold(iii) complexes may undergo electron transfer with the amines (to give amine radical cations) besides producing singlet oxygen for oxidation.

## Conclusions

A series of luminescent Au^III^-encapsulated MOF composites (Au^III^@MOFs) have been synthesized. These Au^III^@MOF solids exhibit long emission lifetimes in air, display solid state two-photon-induced phosphorescence, and function as reusable and size-selective heterogeneous photo-catalysts. The simple approach of incorporating phosphorescent metal complexes with long-lived emissive excited states into MOFs provides a means of developing new classes of heterogeneous photo-functional materials/photo-catalysts with useful applications.

## Supplementary Material

Supplementary information

Crystal structure data
