# Supplementary material for: Metal–organic framework composites with luminescent gold(iii) complexes. Strongly emissive and long-lived excited states in open air and photo-catalysis
Source: Chem Sci. 2015 Sep 21;6(12):7105–11. doi: 10.1039/c5sc02216a (PMC5947532; doi:10.1039/c5sc02216a)
Supplement: Supplementary file 1 [file SC-006-C5SC02216A-s001.pdf]

Electronic Supplementary Material (ESI) for Chemical Science.
This journal is © The Royal Society of Chemistry 2015

Electronic Supplementary Information

Table of contents

Experimental Section S3

Materials and methods S3

Synthesis of MOF1 S3

X-ray single crystal diffraction of MOF1 S4

Synthesis of Au^{III}@MOF1 and Au^{III}@MOF2 composites S4

General procedure for light-induced electron transfer experiments S4

General procedure for two-photon absorption experiments S5

General procedure for the oxidation of secondary amine using Au^{III}@MOF1 (Au^{III} = Au1–Au3), Au^{III} complexes, and MOF1 as catalysts S5

General procedure for size selective catalysis S5

General procedure for investigation of Au4 and Au4@MOF1 in photocatalysis S5

General procedure for oxidative cyanation of tertiary amine using Au1@MOF1, Au1, and MOF1 as catalysts S6

General procedure for oxidative Mannich-type reaction using Au2@MOF2, Au2, and MOF2 as catalysts S6

General procedure for oxidative aza-Henry reaction using Au2@MOF2, Au2, and MOF2 as catalysts S6

General procedure for aerobic oxidative hydroxylation of 4-chlorophenylboronic acid using Au2@MOF2, Au2, and MOF2 as catalysts S6

General procedure for reductive cyclization of alkyl iodide using Au2@MOF2, Au2, and MOF2 as catalysts S6

References S8

Table S1 Molecular sizes of Au^{III} complexes Au1–Au4 S9

Table S2 Contents of Au^{III} complexes in Au^{III}@MOFs as determined by ICP spectroscopy S9

Table S3 Product yields and turnovers for oxidative Mannich-type reaction and oxidative aza-Henry reaction using Au2@MOF2 and Au2 as catalysts S27

Table S4 Aerobic oxidative hydroxylation of 4-chlorophenylboronic acid using Au2@MOF2 and Au2 as catalysts S28

Table S5 Reductive cyclization of alkyl iodide using Au2@MOF2 and Au2 as catalysts S29

Table S6 Reduction potentials $E(\mathbf{Au}^{0/-})$ and excited state reduction potentials $E(\mathbf{Au}^{*}/\mathbf{Au}^{-})$ for Au1–Au4 S30

Table S7 Nanosecond time-resolved transient absorption (TA) measurements of solutions of Au1–Au4 alone and in the presence of dibenzylamine substrate in degassed DMF S31

Table S8 Nanosecond time-resolved transient absorption (TA) measurements of solutions of Au2 alone and in the presence of *N*-phenyl-1,2,3,4-tetrahydroisoquinoline substrate in degassed DMF S34

Fig. S1 Coordination environment of Cd^{2+} ions, connectivity of TATMB ligand, 18-membered rings in MOF1, and topological structure of MOF1 S10

Fig. S2 TGA of MOF1 under nitrogen gas S11

Fig. S3 Nitrogen sorption isotherms of MOF1 S12

Fig. S4 Photographs of MOF1 and MOF2 after being immersed in solutions of Au^{III} complexes S13

Fig. S5 PXRD for MOF1, MOF2, and Au^{III}@MOFs S14

Fig. S6 PL spectra of Au^{III} solutions under degassed and aerobic conditions S15

Fig. S7 Steady-state emission spectra of Au2@MOF2 (8.26 wt% Au2) under Ar or air conditions S16

Fig. S8 Optical microscopy images of Au2@MOF2 and its cross section S17

Fig. S9 SEM-image and EDX Au-elemental mapping of the cross section of Au2@MOF2 S18

Fig. S10 EDX spectrum of Au2@MOF2 (8.26 wt% Au2) S19

Fig. S11 Nitrogen sorption isotherms of MOF2 before and after incorporation of Au2 S20

Fig. S12 Nitrogen sorption isotherms of Au2@MOF2-MV^{2+} composite without sample pretreatment. S21

Fig. S13 PXRD of MOF1 and MOF2 after being immersed in MeCN for 24 h S22

Fig. S14 Time course of light-induced aerobic oxidation of dibenzylamine using Au2@MOF1, Au2, and MOF1 as catalysts S23

Fig. S15 Time course of light-induced aerobic oxidation of dibenzylamine using Au3@MOF1, Au3, and MOF1 as catalysts S24

Fig. S16 Time course of light-induced oxidation of dibenzylamine to imine using Au2@MOF2 (1.43 wt%) and Au2@MOF2 (8.26 wt%) as catalysts S25

Fig. S17 Time course of oxidative cyanation of tertiary amine using Au1@MOF1, Au1, and MOF1 as catalysts S26

Experimental Section

Materials and methods: All reagents and solvents for the syntheses were purchased from commercial sources and used as received, unless otherwise indicated. MOF2[1] and Au^{III} complexes Au1 ($CF_3SO_3^-$ salt),[2d] Au2 ($CF_3SO_3^-$ salt),[2c] Au3 ($CF_3SO_3^-$ salt),[2a] and Au4 (BF_4^- salt)[2b] were synthesized according to the literature procedures. PXRD patterns were recorded on a Siemens D5005 diffractometer with Cu Kα (λ = 1.5418 Å) radiation in the range of 3−50°. Elemental analyses (C, H, and N) were performed on a Perkin-Elmer 240C elemental analyzer. Thermogravimetric analyses (TGA) experiments were performed on a Perkin-Elmer TGA 7 analyzer heated from room temperature to 800 °C under nitrogen gas atmosphere with a heating rate of 10 °C min^{-1}. Steady-state emission spectra were recorded on a SPEX 1681 Fluorolog-3 spectrophotometer. Emission lifetime measurements were performed on a Quanta Ray GCR 150-10 pulsed Nd:YAG laser system. Errors for λ values (±1 nm), τ (±10 %), and Φ (± 10 %) are estimated. Nuclear magnetic resonance spectra were recorded on Bruker DPX-400 or DPX-500 FT-NMR spectrometer with chemical shifts (in ppm) relative to tetramethylsilane or non-deuterated solvent residual. The N_2 sorption measurements were performed on automatic volumetric adsorption equipment (Autosorb-IQ2).

Synthesis of H_3TATMB ligand for MOF1:

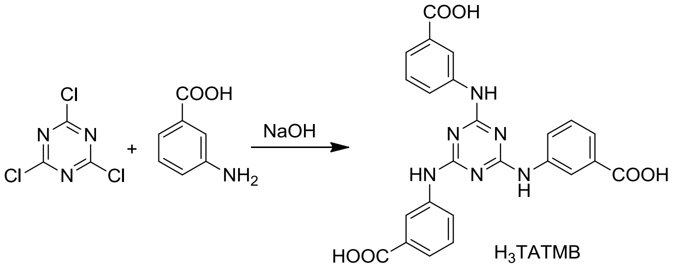

Compound H_3TATMB was synthesized by a procedure similar to the previously reported one for the synthesis of H_6TATPT.[3] Cyanuric chloride (1.84 g, 0.01 mol) was added to H_2O (100 mL) and then was stirred at 0 °C for 30 min. After that a mixture of 3-aminobenzoic acid (1.37 g, 0.01 mol) and NaOH (0.5 g, 0.0125 mol) was added dropwise into the aqueous solution and stirred at ca. 0 °C for 1.5 h. Then a solution of 3-aminobenzoic acid (2.74 g, 0.02 mol) and sodium hydroxide in H_2O (25 mL) was added and the reaction mixture was stirred at room temperature. After stirring at room temperature for 2 h, the mixture was heated slowly to boiling and refluxed for 3 h. During this period, the pH of the mixture was maintained between 10 and 11 by the addition of sodium hydroxide solution. After cooling, the cloudy solution was filtered and the volume of filtrate was increased to approximately 500 mL by adding water (300 mL). The solution was acidified with concentrated hydrochloric acid where upon a crystalline solid precipitated out. The mixture was allowed to cool to room temperature and filtered. After washing thoroughly with cold water, the product was dried in a vacuum oven. Yield: 80%. ^{1}H NMR ([D6]DMSO, 400 MHz): δ = 7.38 (3H), 7.56 (3H), 8.16 (3H), 8.25 (3H), 9.50 (3H), 12.85 (3H); ^{13}C NMR (150 MHz, [D6]DMSO): δ = 167.32, 164.10, 139.93, 131.04, 128.64, 124.81, 122.93, 121.32.

Synthesis of MOF1: A mixture of $Cd(NO_3)_2\cdot 6H_2O$ (0.10 mmol), H_3TATMB (0.12 mmol), and DMF (6 mL) was stirred for 10 min. The mixture was then transferred to, and sealed in, a Teflon

reactor (18 mL) and heated at 120 ℃ for 72 h. After that, the mixture was cooled to room temperature at 5 ℃·h^{-1}. Colorless crystals of MOF1 were obtained (85.8% yield based on TATMB). Elemental analysis (%) calcd for $[Me_2NH_2]_2[(Cd_2(TATMB)_2]\cdot 10DMF\cdot 3H_2O$ ($C_{82}H_{116}N_{24}O_{25}Cd_2$): C, 47.75; H, 5.67; N, 16.30; found: C, 47.68; H, 5.72; N, 16.38.

X-ray single crystal diffraction of MOF1: Diffraction data were recorded on a Bruker Apex CCD II area-detector diffractometer with graphite-monochromated Mo Kα radiation (λ = 0.71073 Å) at 293 K. Absorption corrections were applied using multi-scan technique. The structure was solved by direct method of SHELXS-97 and refined by full-matrix least-square techniques using the SHELXL-97 program. Because guest solvent molecules in channels were highly disordered and could not be modeled properly, the SQUEEZE routine of PLATON was applied to remove contributions to scattering from solvent molecules. The reported refinements are of guest-free structure by SQUEEZE routine. The X-ray crystallographic data have been deposited at the Cambridge Crystallographic Data Centre (CCDC), under deposition number CCDC 993259. These data can be obtained free of charge from The Cambridge Crystallographic Data Centre via www.ccdc.cam.ac.uk/data_request/cif.

Synthesis of Au^{III}@MOF1 composites: MOF1 (20 mg) was suspended in 5 mL of DMF solutions containing 4 × 10^{-6} mol of Au^{III} complexes (Au1−Au4) under stirring in 10-mL sealed glass vials. After 1.5, 3, 3, and 2 days, the immersed samples were taken out and washed with DMF (~4 × 10 mL) until the washings were colorless (to remove residual Au^{III} complex on the surface). The concentrations of encapsulated Au^{III} complexes in composited materials were measured by ICP spectroscopy and the results are shown in Table S2.

Synthesis of Au^{III}@MOF2 composites: MOF2 (20 mg) was suspended in 5 mL of DMF solutions containing 5 × 10^{-6} mol of Au1 or Au2 under stirring in 10-mL sealed glass vials. After 5 and 7 days, the immersed samples were taken out and washed with fresh DMF (~4 × 10 mL) until the washings were colorless (to remove residual Au^{III} complex on the surface). ICP spectroscopy was employed to measure the concentrations of encapsulated Au^{III} complexes (Table S2).

General preparation of solids in argon and in degassed solutions for emission and lifetime measurements: Solid samples of composited materials in argon were prepared firstly in a quartz tube using a high vacuum line and then argon was introduced into the tube. These procedures were repeated five times and the tube filled with argon was sealed during the photophysical studies. Solutions for photophysical studies were degassed by using a high vacuum line in a two-compartment cell with five freeze-pump-thaw cycles.

General procedure for light-induced electron transfer experiments: Fresh sample of Au2@MOF2 (20 mg, 1.43 wt% Au2) was immersed into the solution of methyl viologen dication (0.01 mmol) in DMF (2 mL) at room temperature for 12 h. The prepared sample was filtered and washed with fresh DMF (~2 × 10 mL). When the sample was dry, it was put into a test tube and a drop of triethylamine was dripped into the wall of the tube. After a few minutes, this tube was irradiated by a 300 W xenon lamp for several seconds with the emergence of blue color. After heating for a few minutes, the blue color disappeared. Recycle experiment was implemented by dripping triethylamine into the tube. Control experiment was set through irradiated mixed solution

of Au2 (2×10^{-5} mol L^{-1}), methyl viologen dication (0.01 mmol), and triethylamine under the same conditions.

General procedure for two-photon absorption experiments: Solid sample of Au3@MOF1 (20 mg, 2.34 wt% Au3) kept in a 11 cm-tall quartz cell was degassed using a high vacuum line and then argon was introduced into the cell. These procedures were repeated five times and the cell filled with argon was sealed during the two-photon absorption experiments. The experiments were performed under the excitation of 756 nm laser which was generated from a self-mode-locked Ti:sapphire oscillator (Tsunami) pumped by a 10 W solid-state laser diode (Millennia). It has a ~80 fs pulse width and a repetition rate of 82 MHz. The wavelength of the laser beam is tunable between 700 to 860 nm. Emission signals from the sample were collected by a pair of lenses, analysed by a monochromator (Acton SP305), and detected with a PMT (Hamamatsu R928). The laser power for the experiments is ~3.0 W. The diameter of the laser beam is 3 mm.

General procedure for the oxidation of secondary amine using Au^{III}@MOF1 (Au^{III} = Au1–Au3), Au^{III} complexes, and MOF1 as catalysts: The procedure is described with Au^{III} = Au1 as a typical example. Au1@MOF1 (20 mg, 1.70 wt% Au1), Au1 solution (2×10^{-7} mol), or MOF (20 mg) was put in a test tube, and then dibenzylamine (0.107 mmol) in acetonitrile (1.6 mL) was added into the test tube. The reaction mixture was bubbled with solvent-saturated oxygen gas throughout the experiment (1 atm) and irradiated at $\lambda > 400$ nm with a 300 W xenon lamp as the light source at room temperature. After irradiating for 2 h, 2–3 μL of solution was taken out and injected into a gas chromatography (GC) with FID detector. The product yield was calculated based on GC detection. For reaction using Au1@MOF1 as catalyst, every 2 h, the product turnover was determined by GC and additional 0.107 mmol of dibenzylamine was added into the solution. The recycle of heterogeneous catalyst Au1@MOF1 (Fig. 5c in text) was performed by washing the catalyst with acetonitrile (4 × 1 mL) in the test tube and after the final wash, fresh dibenzylamine was added.

General procedure for size selective catalysis: Competition oxidation reactions were set up using equimolar amounts of secondary amines (S1: 0.053 mmol, S2: 0.053 mmol) in THF (1.6 mL) for both catalysts Au1 (2×10^{-6} mol) and Au1@MOF1 (10 mg, 1.70 wt% Au1). The reaction mixture was bubbled with oxygen gas throughout the experiment (1 atm) and irradiated at $\lambda > 400$ nm (with a 300 W Xenon lamp as the light source) at room temperature for 1.5 h. The product yield ratio was determined by ^{1}H NMR spectroscopy.

General procedure for investigation of Au4 and Au4@MOF1 in photocatalysis: Photooxidation of secondary amines was set as a model reaction for this investigation. Au4@MOF1 (8 mg, 4.50 wt% Au4), Au4 (6×10^{-7} mol), or Au4 (6×10^{-6} mol) was put in a test tube, and then dibenzylamine (0.107 mmol) in acetonitrile (1.6 mL) was added into the test tube. The reaction mixture was bubbled with solvent-saturated oxygen gas throughout the experiment (1 atm) and irradiated at $\lambda > 400$ nm (with a 300 W Xenon lamp as the light source) at room temperature. After irradiating for 0.5 h interval, 2–3 μL of the solution was taken out and injected into a gas chromatography (GC) with FID detector. The product yield was calculated based on GC detection. The recycle of heterogeneous catalyst Au4@MOF1 was performed by washing the catalyst with acetonitrile (4 × 1 mL) in the test tube, and after the final wash, fresh dibenzylamine

was added.

General procedure for oxidative cyanation of tertiary amine using Au1@MOF1, Au1, and MOF1 as catalysts: Au1@MOF1 (10 mg, 1.70 wt% Au1), Au1 solution (1×10^{-7} mol), or MOF (10 mg) was put in a test tube, and then 2-phenyl-1,2,3,4-tetrahydroisoquinoline (0.053 mmol, 11 mg) and trimethylsilanecarbonitrile (Me_3SiCN, 13 μL) mixed in acetonitrile solution (1.6 mL) were added. The reaction mixture was bubbled with solvent-saturated oxygen gas throughout the experiment (1 atm) and irradiated at λ > 400 nm (with a 300 W Xenon lamp as the light source) at room temperature. For reaction using Au1@MOF1 as catalyst, the progress of reaction was monitored by TLC. When almost all substrate was consumed, the solution was subjected to ^{1}H NMR spectroscopic analysis. Then, equivalent amounts of substrate and Me_3SiCN were added and irradiation started again (within 10 h, 0.371 mmol of 2-phenyl-1,2,3,4-tetrahydroisoquinoline and 91 μL of Me_3SiCN were added). The product yield (based on conversion) was determined by ^{1}H NMR spectroscopy.

General procedure for oxidative Mannich-type reaction using Au2@MOF2, Au2, and MOF2 as catalysts: Au2@MOF2 (20 mg, 1.43 wt% Au2), Au2 (6.4×10^{-7} mol), or MOF2 (20 mg) was put in a test tube. 2-Phenyl-1,2,3,4-tetrahydroisoquinoline (0.128 mmol × 2) in 0.8 mL of mixed solvent of acetone and MeCN (1:1 v/v) was added into the test tube, followed by addition of L-proline (0.02 mmol) in methanol (0.8 mL). The reaction mixture was bubbled with solvent-saturated oxygen gas throughout the experiment (1 atm) and irradiated at λ > 370 nm (with a 300 W Xenon lamp as the light source) at room temperature. The product yield was determined by ^{1}H NMR spectroscopy using 4,4′-dimethyl-2,2′-bipyridine as internal standard.

General procedure for oxidative aza-Henry reaction using Au2@MOF2, Au2, and MOF2 as catalysts: Au2@MOF2 (20 mg, 1.43 wt% Au2), Au2 (5×10^{-7} mol), or MOF2 (20 mg) was put in a test tube. 2-Phenyl-1,2,3,4-tetrahydroisoquinoline (0.1 mmol × 2) in $MeNO_2$ (3 mL) was added into the test tube. The reaction mixture was bubbled with solvent-saturated oxygen gas throughout the experiment (1 atm) and irradiated at λ > 370 nm (with a 300 W Xenon lamp as the light source) at room temperature. The product yield was determined by ^{1}H NMR spectroscopy using 4,4′-dimethyl-2,2′-bipyridine as internal standard.

General procedure for aerobic oxidative hydroxylation of 4-chlorophenylboronic acid using Au2@MOF2, Au2, and MOF2 as catalysts: Au2@MOF2 (20 mg, 1.43 wt%), Au2 (5×10^{-7} mol), or MOF2 (20 mg) was put in a test tube. (4-Chlorophenyl)boronic acid (0.3 mmol), *N*,*N*-diisopropylethylamine (0.6 mmol) in DMF (3 mL) were added into the test tube. The reaction mixture was bubbled with solvent-saturated oxygen gas throughout the experiment (1 atm) and irradiated at λ > 370 nm (with a 300 W Xenon lamp as the light source) at room temperature. After the light irradiation, the reaction mixture was cooled to 0 °C, quenched by aqueous solution of HCl, and then extracted with Et_2O. The organic layer was dried over $MgSO_4$. The product yield was determined by ^{1}H NMR spectroscopy using 4,4′-dimethyl-2,2′-bipyridine as internal standard.

General procedure for reductive cyclization of alkyl iodide using Au2@MOF2, Au2, and MOF2 as catalysts: Au2@MOF2 (20 mg, 1.43 wt%), Au2 (5×10^{-7} mol), or MOF2 (20 mg) was put in a test tube. Diethyl 2-allyl-2-(3-iodopropyl)malonate (0.1 mmol),

N,*N*-diisopropylethylamine (0.2 mmol) in MeCN (2 mL) were added into the tube. After being degassed by bubbling of argon, the reaction mixture was irradiated at λ > 370 nm (with a 300 W Xenon lamp as the light source) at room temperature. The product yield was determined by ^{1}H NMR spectroscopy using 4,4′-dimethyl-2,2′-bipyridine as internal standard.

References

1 C.-Y. Sun, C. Qin, C.-G. Wang, Z.-M. Su, S. Wang, X.-L. Wang, G.-S. Yang, K.-Z. Shao, Y.-Q. Lan and E.-B. Wang, *Adv. Mater.*, 2011, **23**, 5629-5632.

2 (*a*) W.-P. To, K. T. Chan, G. S. M. Tong, C. Ma, W.-M. Kwok, X. Guan, K.-H. Low, C.-M. Che, *Angew. Chem. Int. Ed.*, 2013, **52**, 6648-6652; (*b*) H.-M. Ko, K. K.-Y. Kung, J.-F. Cui, M.-K. Wong, *Chem. Commun.*, 2013, **49**, 8869-8871; (*c*) W.-P. To, G. S.-M. Tong, W. Lu, C. Ma, J. Liu, A. L.-F. Chow, C.-M. Che, *Angew. Chem. Int. Ed.*, 2012, **51**, 2654-2657; (*d*) J. J. Yan, A. L.-F. Chow, C.-H. Leung, R. W.-Y. Sun, D.-L. Ma, C.-M. Che, *Chem. Commun.*, 2010, **46**, 3893-3895.

3 C.-Y. Sun, X.-L. Wang, X. Zhang, C. Qin, P. Li, Z.-M. Su, D.-X. Zhu, G.-G. Shan, K.-Z. Shao, H. Wu and J. Li, *Nat. Commun.*, 2013, **4**, 2717-2724.

Table S1 Molecular sizes of Au^{III} complexes Au1–Au4.

Au^{III} complex	Molecular size ($Å^3$)[a]
Au1	6.1 × 11.1 × 12.1
Au2	6.1 × 11.1 × 14.9
Au3	13.5 × 15.1 × 15.3
Au4	6.8 × 11.5 × 11.6

[a] Determined by Materials Studio v 5.0 (*Materials Studio v 5.0*, Accelrys Software Inc., San Diego, CA 92121, USA) combining with CCDC data.

Table S2 Contents of Au^{III} complexes in Au^{III}@MOFs as determined by ICP spectroscopy.

Au^{III}@MOF	Content of Au^{III} complex (wt%)
Au1@MOF1	1.70
Au1@MOF2	2.73
Au2@MOF1	0.91
Au2@MOF2	1.43
Au2@MOF2[a]	8.26
Au3@MOF1	2.34
Au4@MOF1	4.50

[a] Obtained by treatment of MOF2 with a 5×10^{-3} M solution of Au2 in DMF.

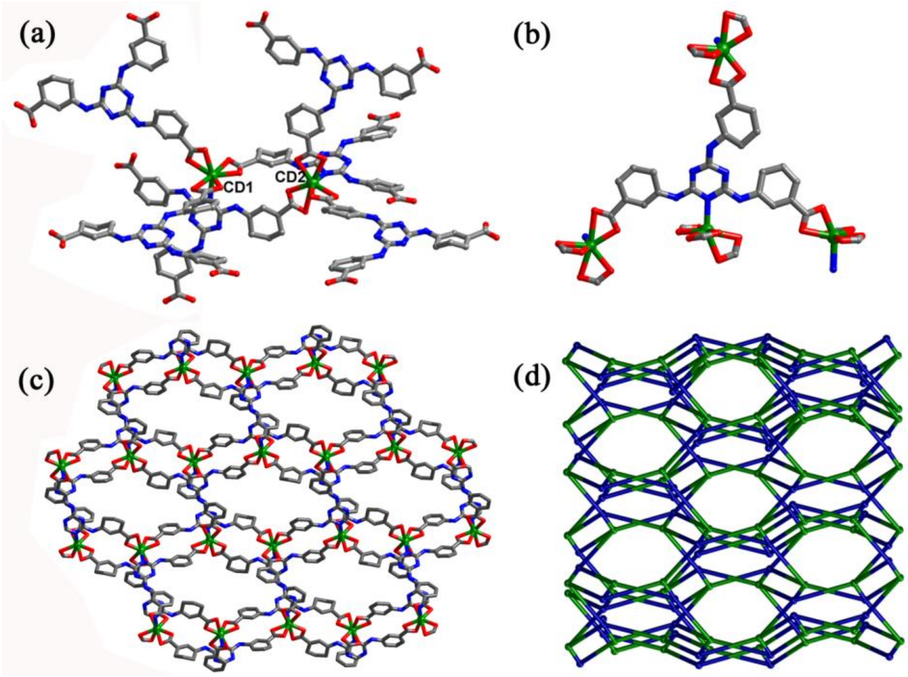

Fig. S1 (a) Coordination environment of Cd^{2+} ions; (b) connectivity of TATMB ligand (C grey, O red, N blue, Cd green); (c) 18-membered rings in MOF1; (d) topological structure of MOF1.

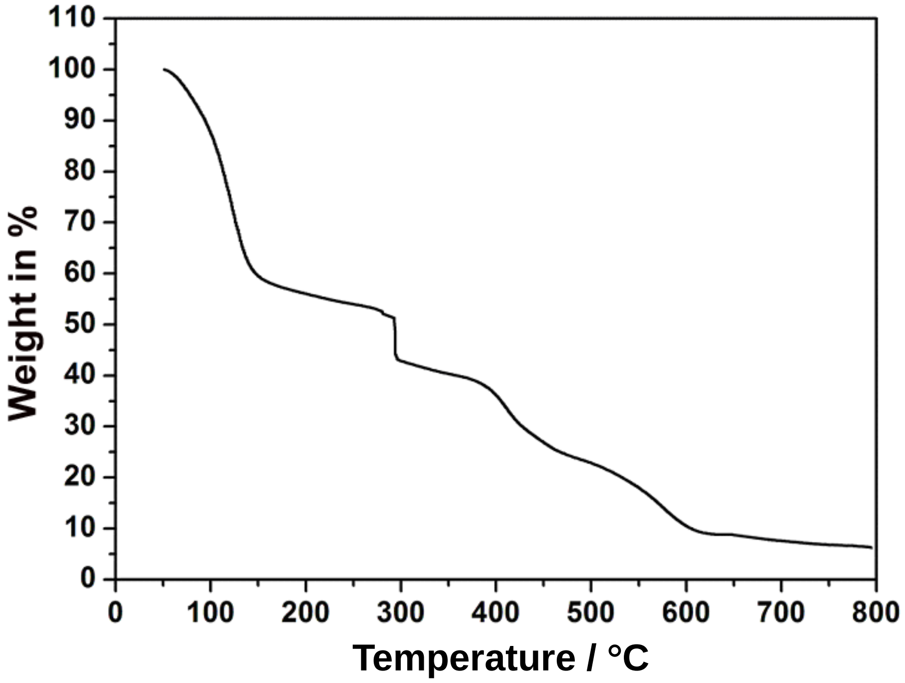

Fig. S2 TGA of MOF1 under nitrogen gas.

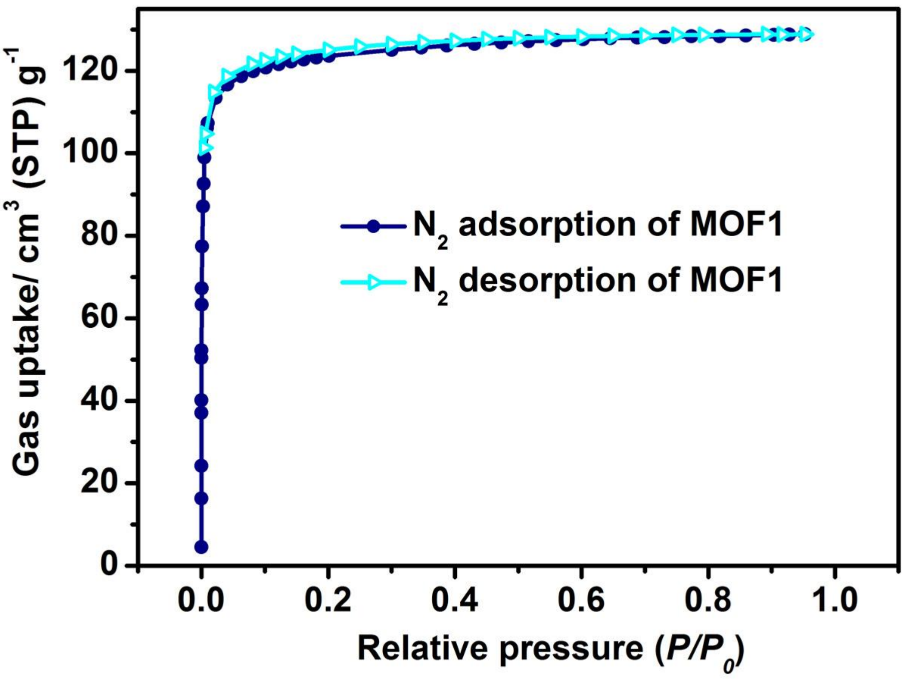

Fig. S3 Nitrogen sorption isotherms of MOF1. The experiments showed that the pore volume of MOF1 is 0.162 cm^3 g^{-1} and BET surface area is 381 m^2 g^{-1}.

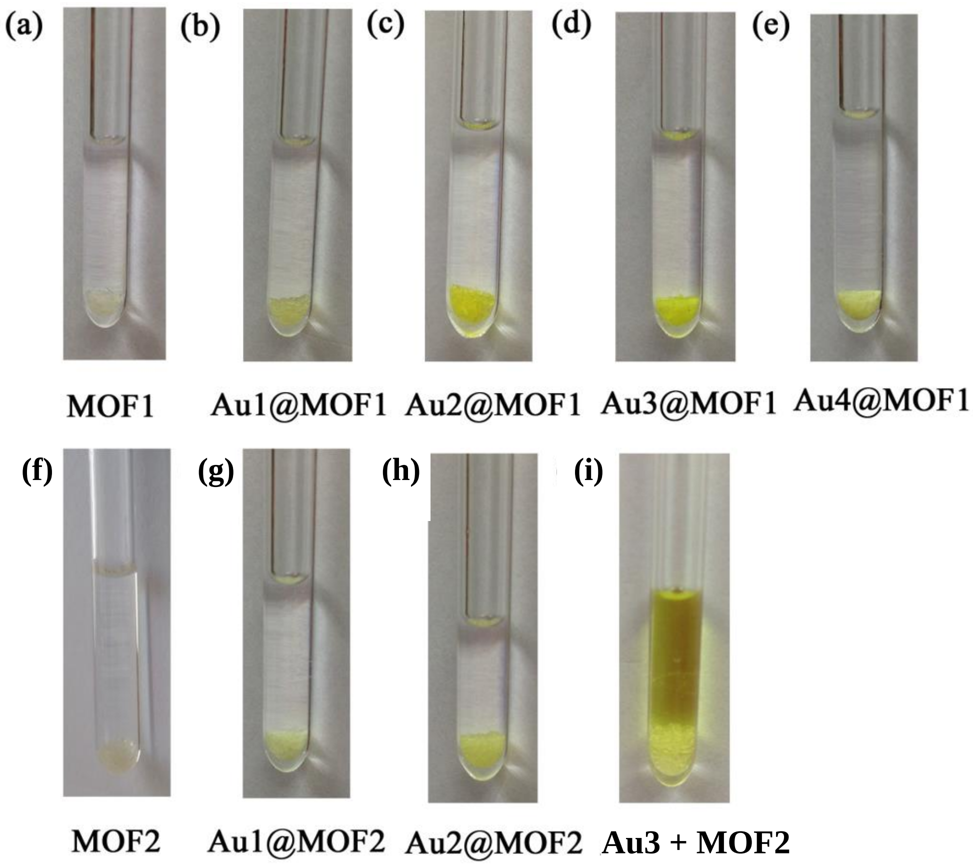

Fig. S4 Photographs of MOF1 and MOF2 immersed in DMF solvent (a and f), MOF1 immersed in solutions of Au1 (b), Au2 (c), Au3 (d), Au4 (e) after 1.5–3 days, and MOF2 immersed in solutions of Au1 (g), Au2 (h), Au3 (i) after 5–7 days.

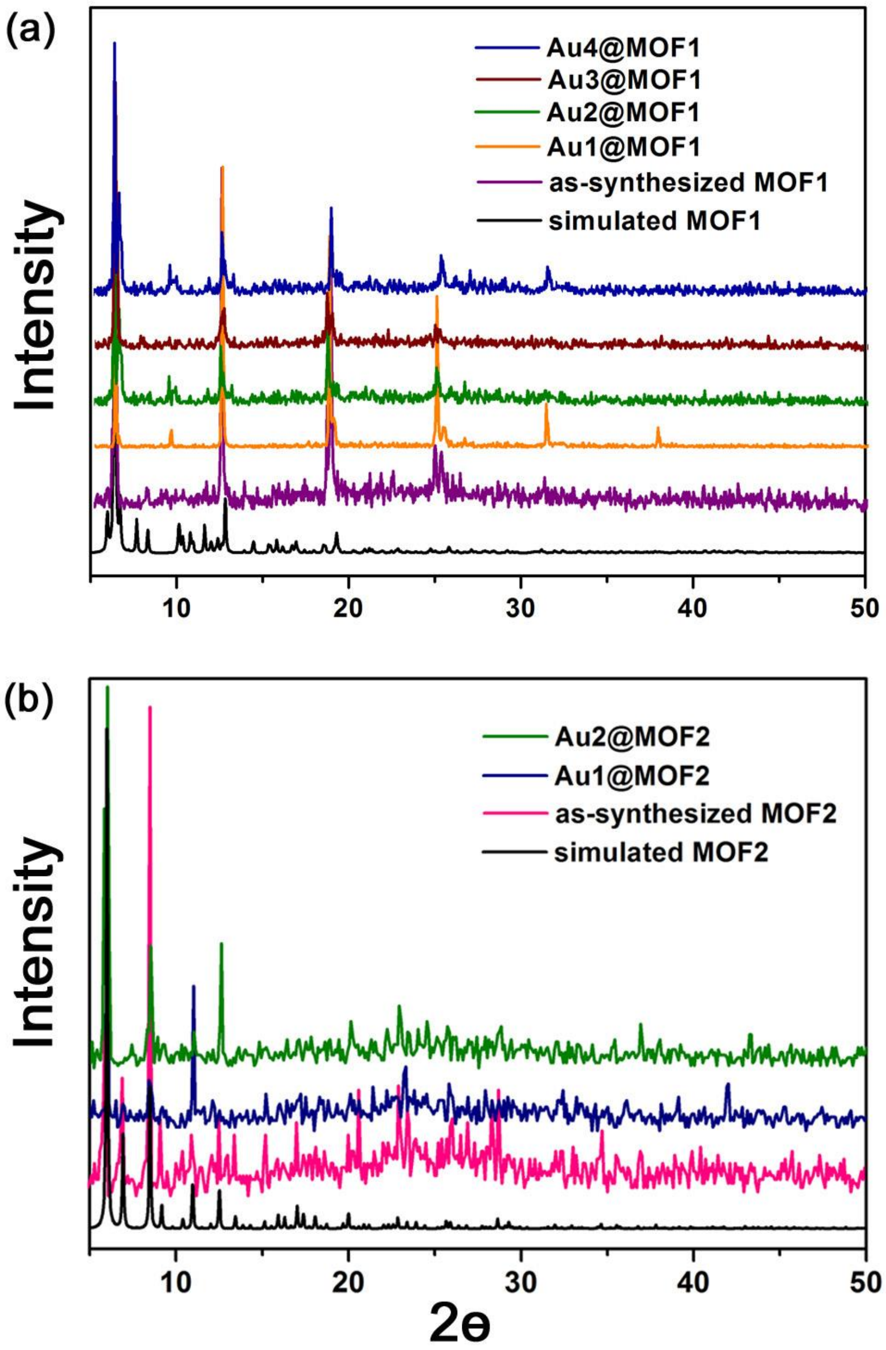

Fig. S5 PXRD of MOF1, MOF2, and Au^{III}@MOFs.

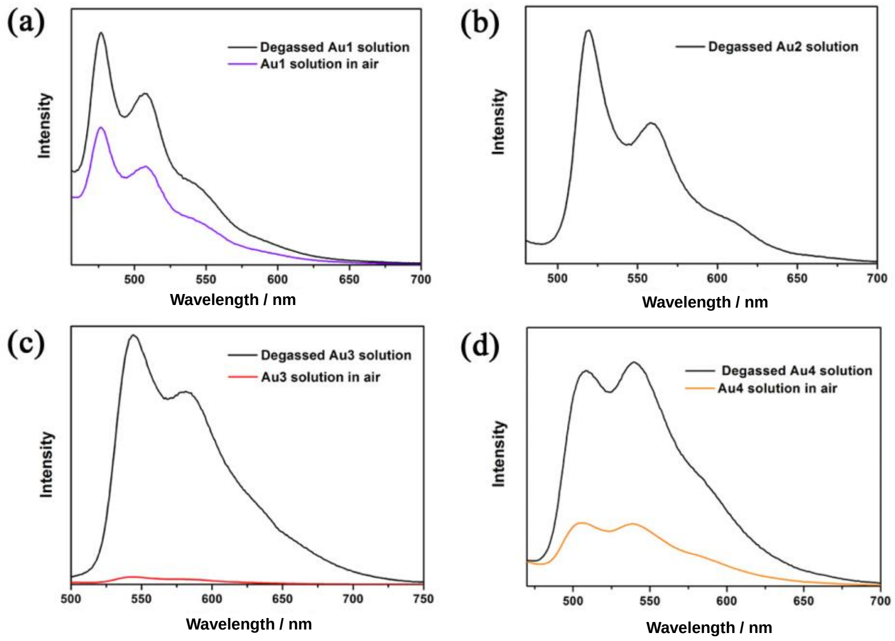

Fig. S6 PL spectra of Au^{III} solutions under degassed and aerobic conditions. For complex Au2, its emission could hardly be recorded in DMF under aerobic conditions.

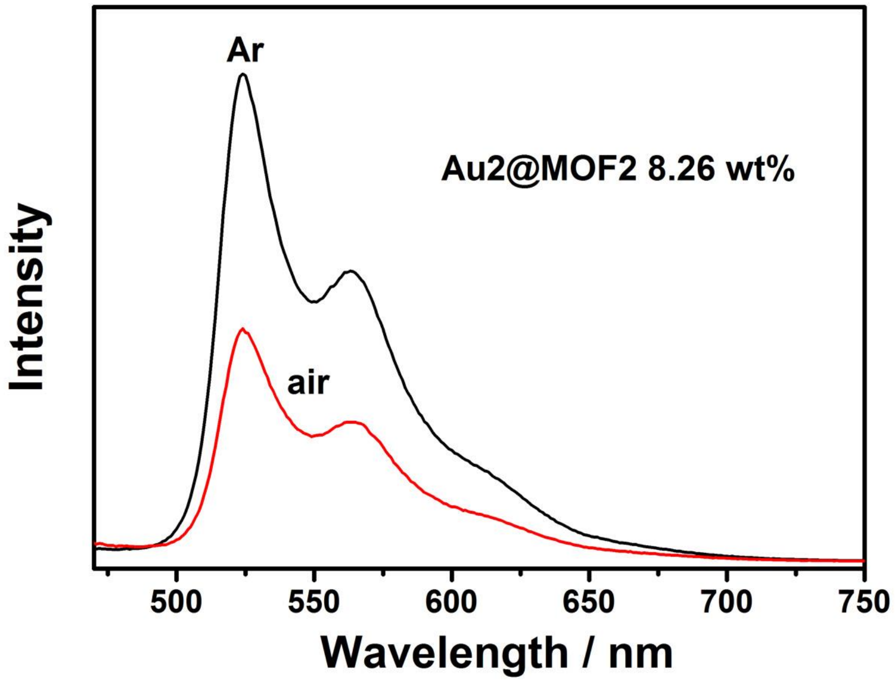

Fig. S7 Steady-state emission spectra of Au2@MOF2 (8.26 wt% Au2) under Ar or air conditions upon excitation at λ = 365 nm at room temperature. The spectra depicted in Fig. 2d in the main text were obtained for Au2@MOF2 (1.43 wt% Au2).

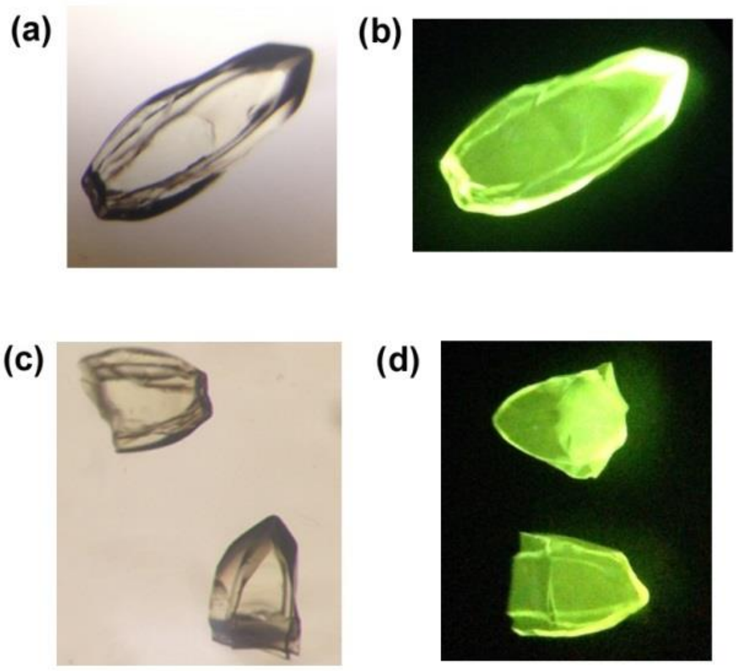

Fig. S8 Optical microscopy images under natural light (a, c) and irradiated by 365 nm UV light (b, d) for a freshly prepared Au2@MOF2 crystal (a, b) and two pieces of the crystal split by a needle (c, d).

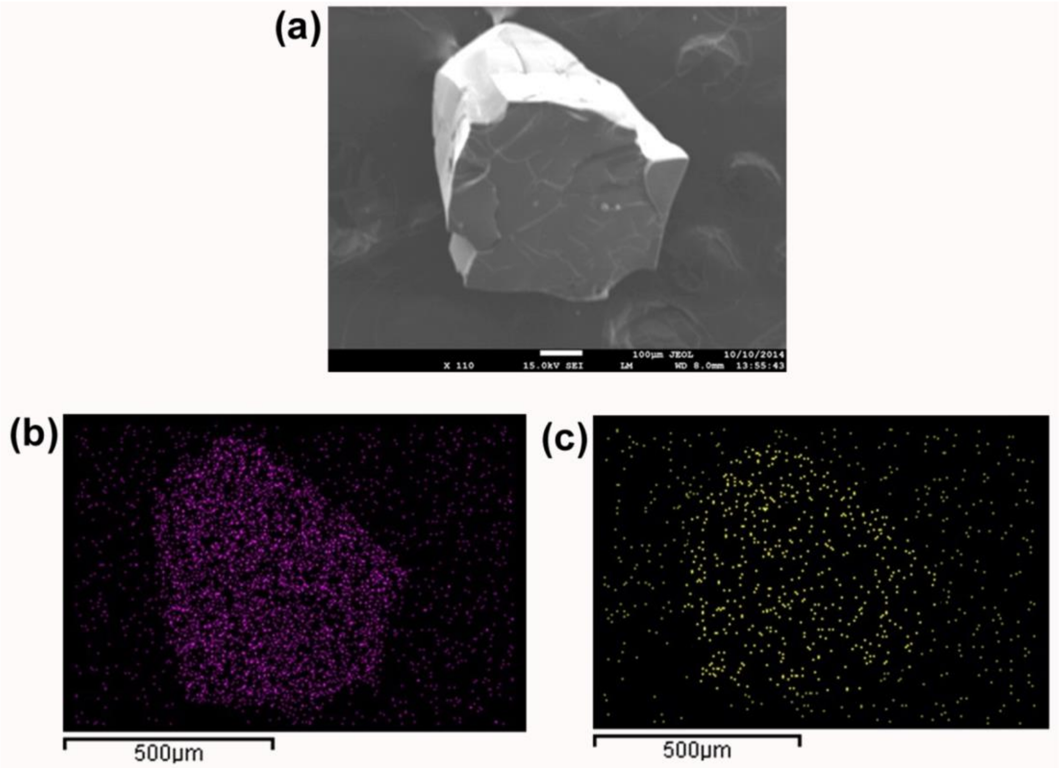

Fig. S9 SEM and EDX studies for Au2@MOF2 crystal using one of two split pieces depicted in Fig. S6. (a) SEM-image coated with carbon; (b) EDX Zn(pink)-elemental mapping; (c) EDX Au(yellow)-elemental mapping.

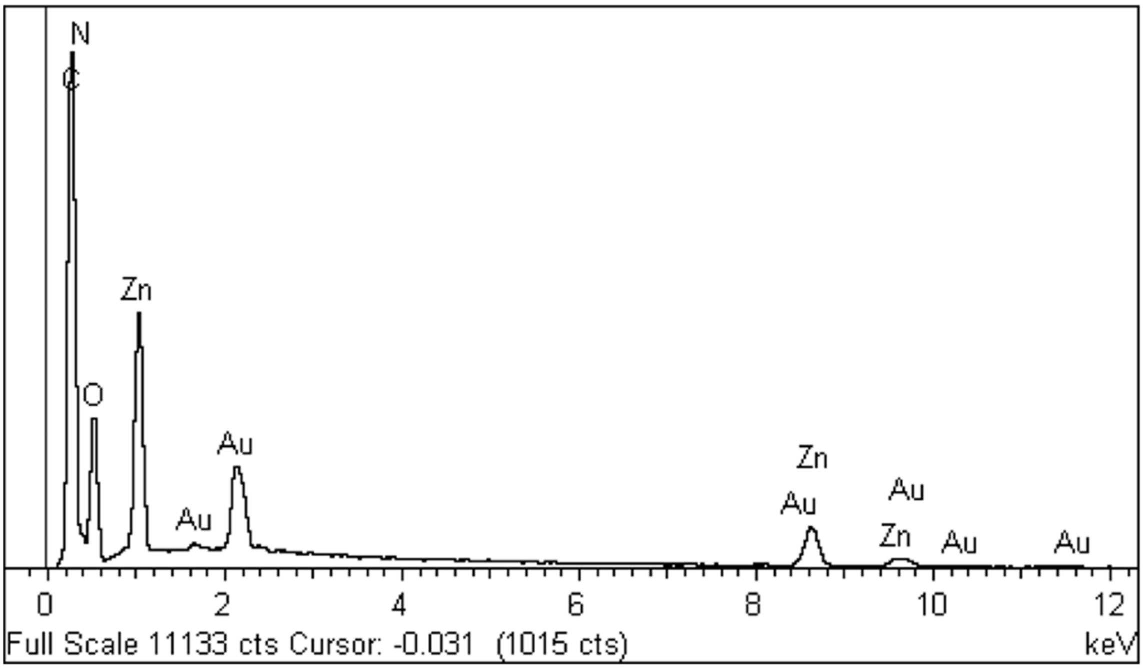

Fig. S10 EDX spectrum of Au2@MOF2 (8.26 wt% Au2). The spectrum was measured using an uncoated sample.

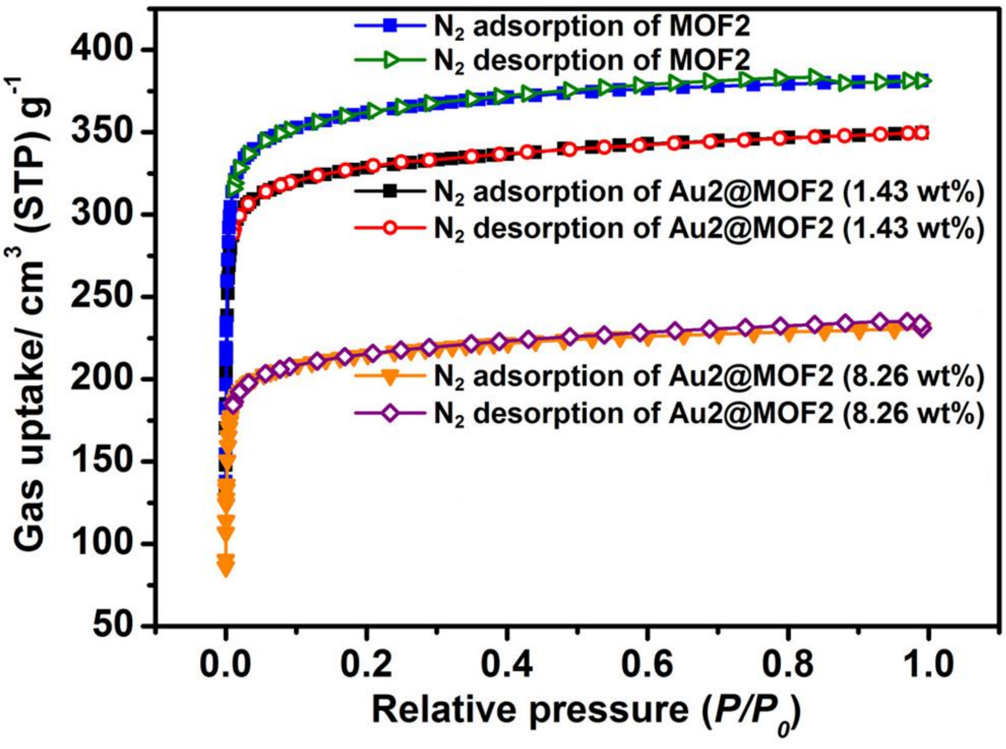

Fig. S11 Nitrogen sorption isotherms of MOF2 before and after incorporation of Au2 to form Au2@MOF2. Results for two samples of Au2@MOF2, with Au2 contents of 1.43 and 8.26 wt% respectively, are depicted. The experiments showed that, upon increasing the Au2 content of Au2@MOF2 from 1.43 wt% to 8.26 wt%, the pore volume decreased from 0.276 to 0.183 $cm^3\ g^{-1}$ with decrease of BET surface area from 1011 to 656 $m^2\ g^{-1}$. The original pore volume and BET surface area of host MOF2 are 0.303 $cm^3\ g^{-1}$ and 1112 $m^2\ g^{-1}$, respectively.

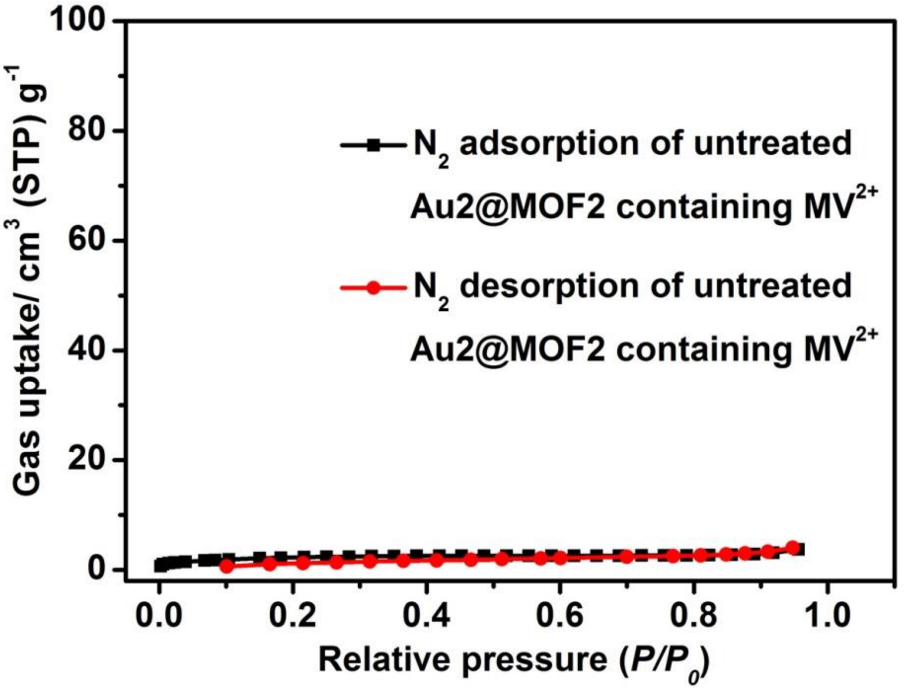

Fig. S12 Nitrogen sorption isotherms of Au2@MOF2-MV^{2+} composite without sample pretreatment. The experiments revealed a small BET surface area of 6.3 m^2 g^{-1}.

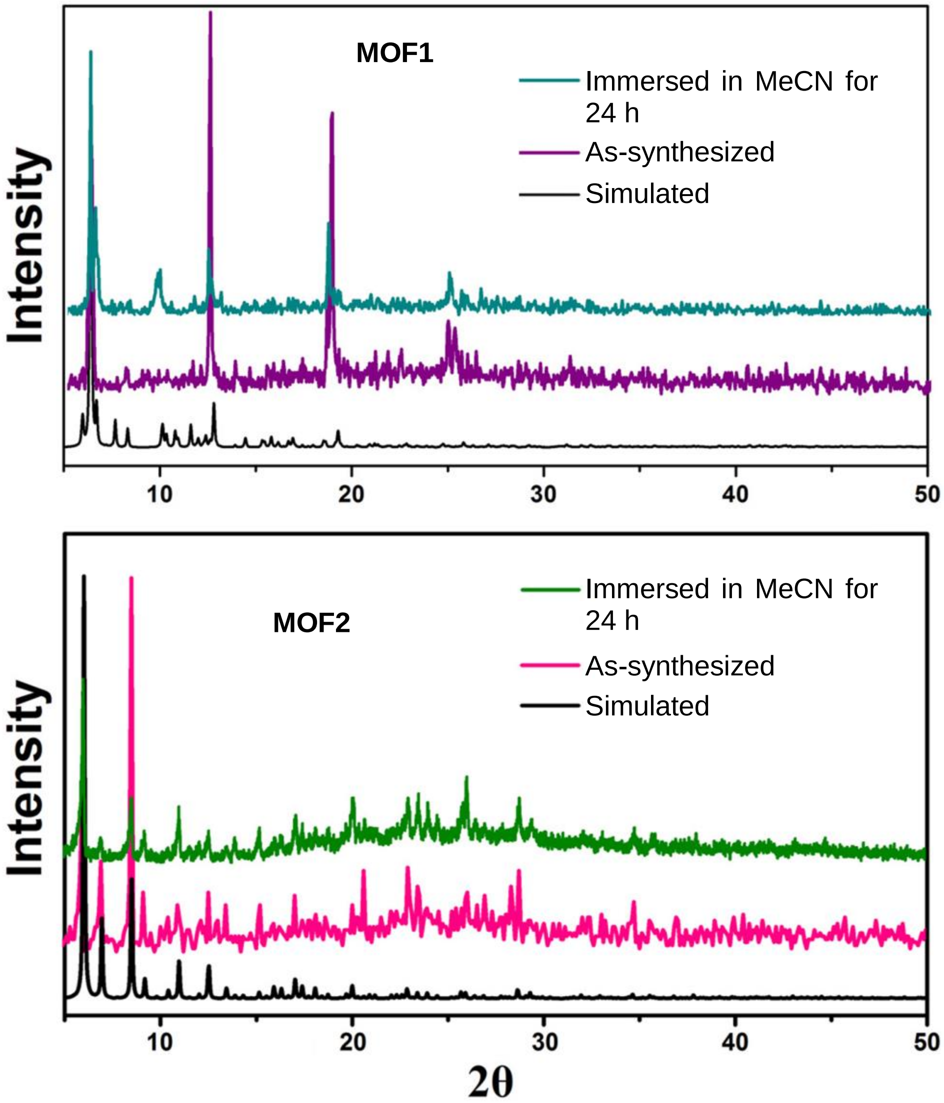

Fig. S13 PXRD of MOF1 (top) and MOF2 (bottom) after being immersed in MeCN for 24 h. For comparison, the corresponding PXRD of as-synthesized MOFs and simulated PXRD are shown.

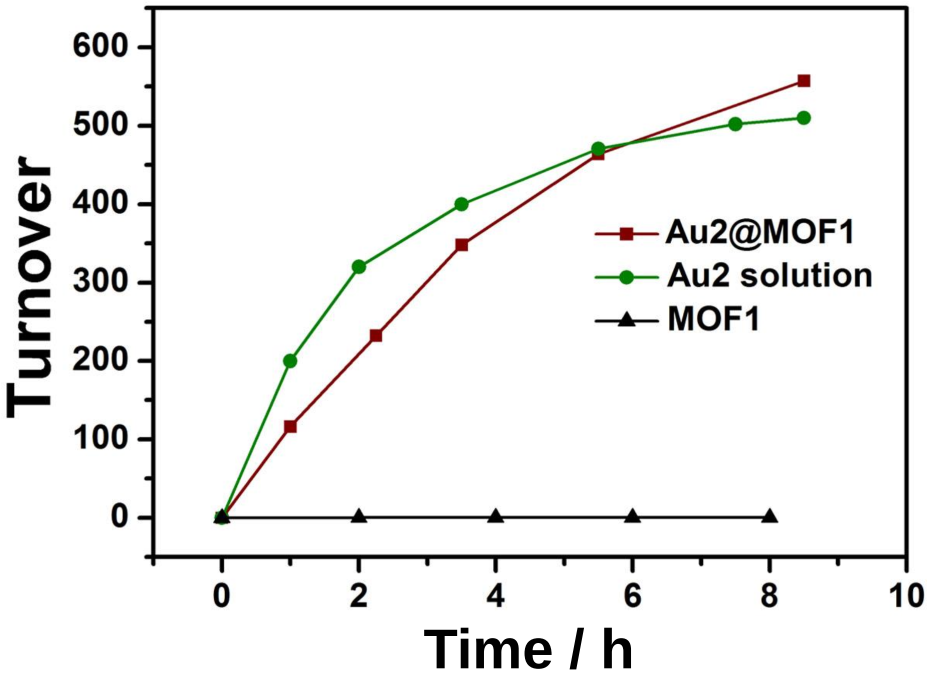

Fig. S14 Time course of light-induced aerobic oxidation of dibenzylamine using Au2@MOF1, Au2, and MOF1 as catalysts.

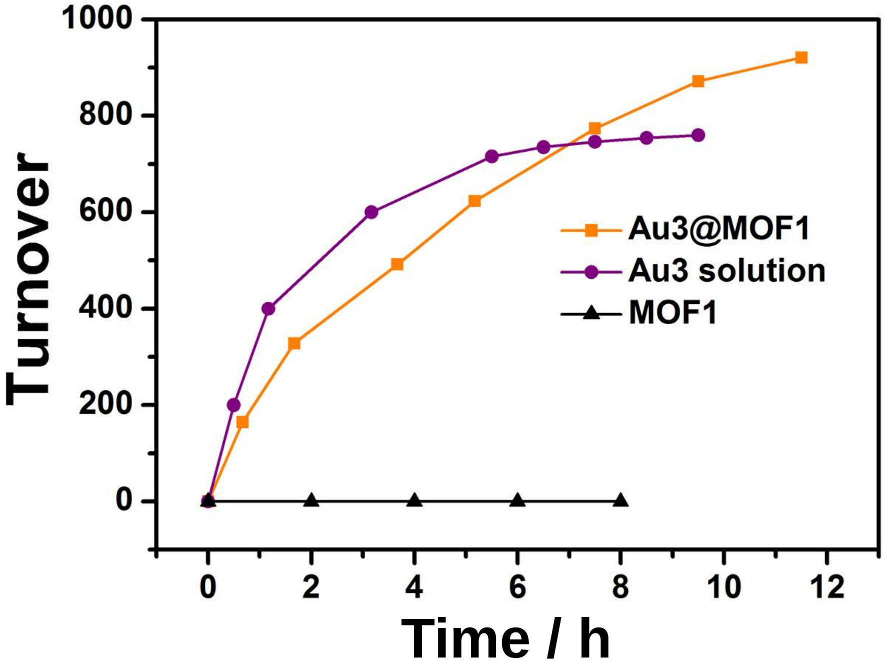

Fig. S15 Time course of light-induced aerobic oxidation of dibenzylamine using Au3@MOF1, Au3, and MOF1 as catalysts.

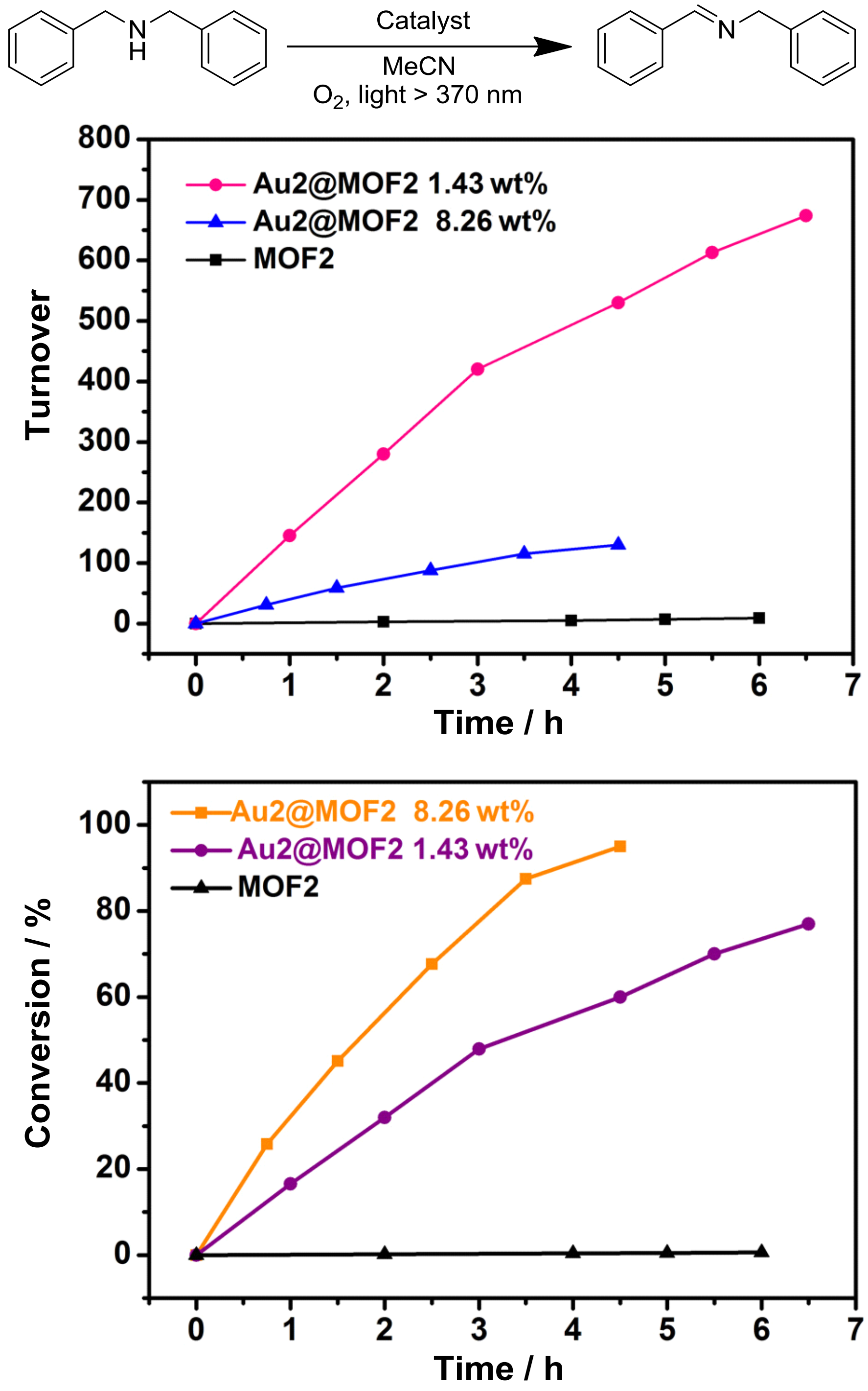

Fig. S16 Time course of light-induced oxidation of dibenzylamine to imine using Au2@MOF2 (1.43 wt% Au2) and Au2@MOF2 (8.26 wt% Au2) as catalysts.

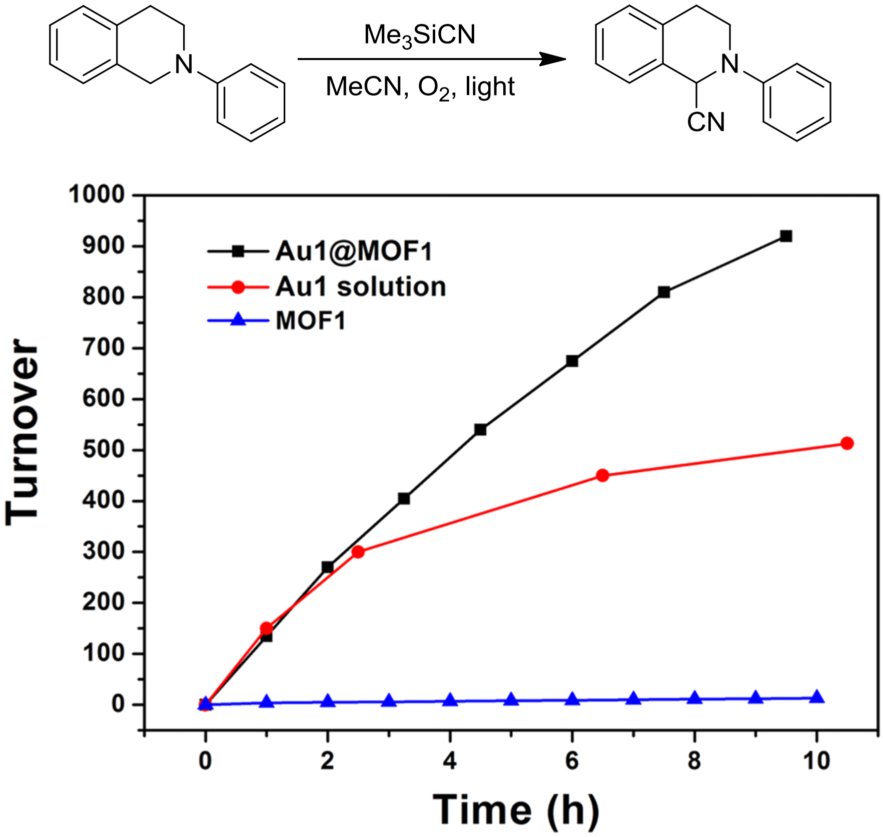

Fig. S17 Time course of oxidative cyanation of tertiary amine using Au1@MOF1, Au1, and MOF1 as catalysts.

Table S3 Product yields and turnovers for oxidative Mannich-type reaction and oxidative aza-Henry reaction using Au2@MOF2 (1.43 wt% Au2) and Au2 as catalysts.

Photochemical reactions	Time (h)	Au2 complex			Au2@MOF2		
		Conver-sion (%)	Yield (%)	TON	Conver-sion (%)	Yield (%)	TON
Oxidative Mannich-type reaction	2.5	>99	73.8	295	90.6	84.2	469
Oxidative aza-Henry reaction	3.5	91.2	83.9	336	90.0	86.1	374

Table S4 Aerobic oxidative hydroxylation of 4-chlorophenylboronic acid using Au2@MOF2 (1.43 wt% Au2) and Au2 as catalysts.[a]

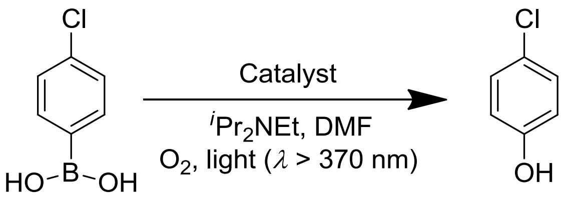

Catalyst	Time (h)	TON	Conversion (%)	Yield (%)
Au2	2	350		
	5	488	87.0	81.4
Au2@MOF2	2	273		
	5	549	85.0	84.3

[a] Control experiment using MOF2 as catalyst resulted in < 3% conversion (reaction time: 5 h).

Table S5 Reductive cyclization of alkyl iodide using Au2@MOF2 (1.43 wt% Au2) and Au2 as catalysts.[a]

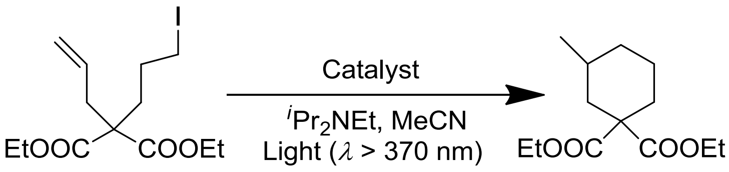

Catalyst	Time (h)	Conversion (%)	Yield (%)	TON
Au2	8	87.1	51.4	103
Au2@MOF2	8	84.7	63.0	137

[a] Control experiment using MOF2 as catalyst resulted in < 3% conversion (reaction time: 8 h).

Table S6 Reduction potentials $E(\mathbf{Au}^{0/-})$ and excited state reduction potentials $E(\mathbf{Au}^{*}/\mathbf{Au}^{-})$ for Au1–Au4.

	$E(\mathbf{Au}^{0/-})$ (V *vs* $Cp_2Fe^{+/0}$)[a]	$E_{0\text{-}0}$ (V)	$E(\mathbf{Au}^{*}/\mathbf{Au}^{-})$[b] (V *vs* $Cp_2Fe^{+/0}$)
Au1	−1.69	2.68	+0.99
Au2	−1.74	2.48	+0.74
Au3	−1.64	2.39	+0.75
Au4	−1.18	2.56	+1.38

[a] Cyclic voltammetry was performed at room temperature (after purging with nitrogen) using PAR potentiostat 273A (equipped with PowerSuite program) and conventional two compartment electrolytic cell. Scan rate: 100 mV s^{-1}. Supporting electrolyte: 0.1 M tetrabutylammonium hexafluorophosphate in DMF. Working electrode: glassy carbon; counter electrode: platinum wire; reference electrode: $Ag/AgNO_3$ (0.1 M in MeCN). Internal standard: ferrocenium/ferrocene couple ($E[Cp_2Fe^{+/0}]$ = +0.04 V). [b] Estimated from the onset of the triplet emission. [c] $E(\mathbf{Au}^{*}/\mathbf{Au}^{-}) = E(\mathbf{Au}^{0/-}) + E_{0\text{-}0}(\mathbf{Au})$.

Table S7 Nanosecond time-resolved transient absorption (TA) measurements of solutions of Au1–Au4 alone and in the presence of dibenzylamine substrate in degassed DMF.[a]

Complex	Transient absorption spectra of complex in degassed DMF	Transient absorption spectra of complex with 0.1 M dibenzylamine in degassed DMF	Decay of transient absorption intensity at specified wavelength
Au1	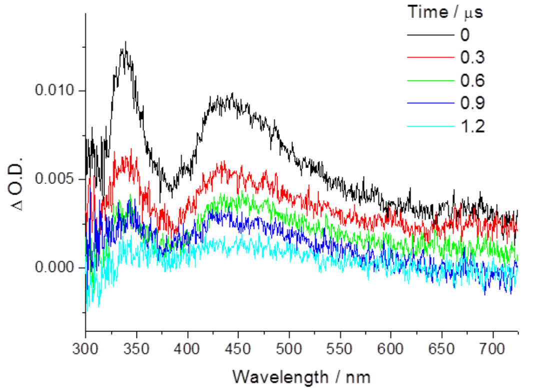	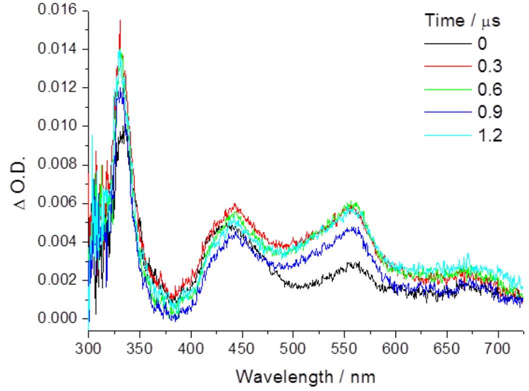	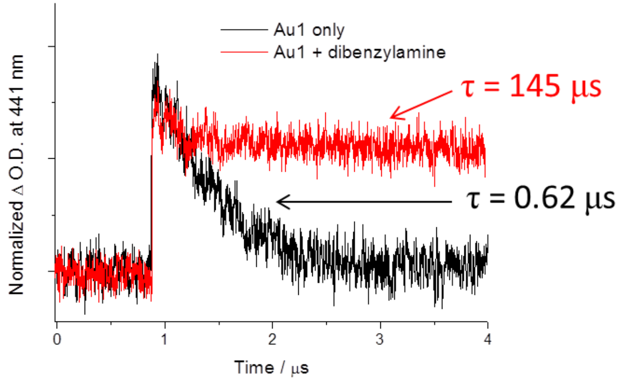
Au2	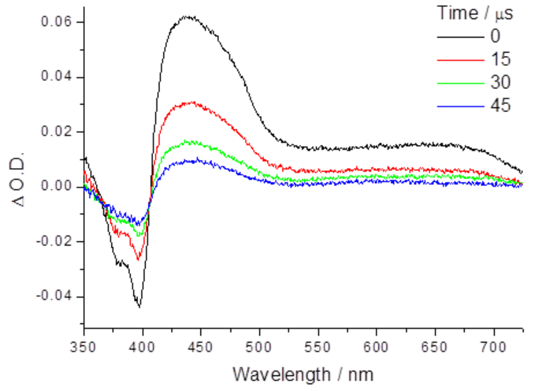	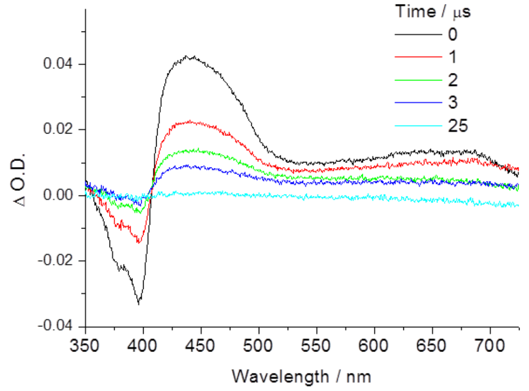	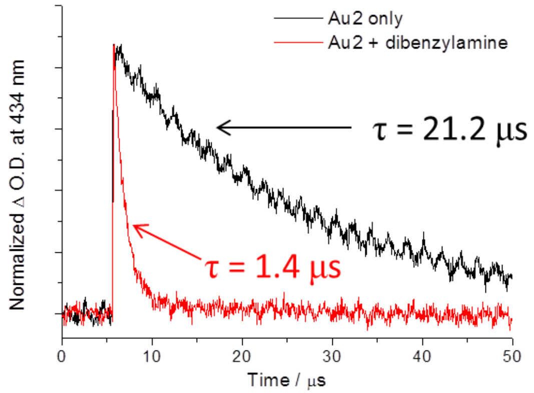

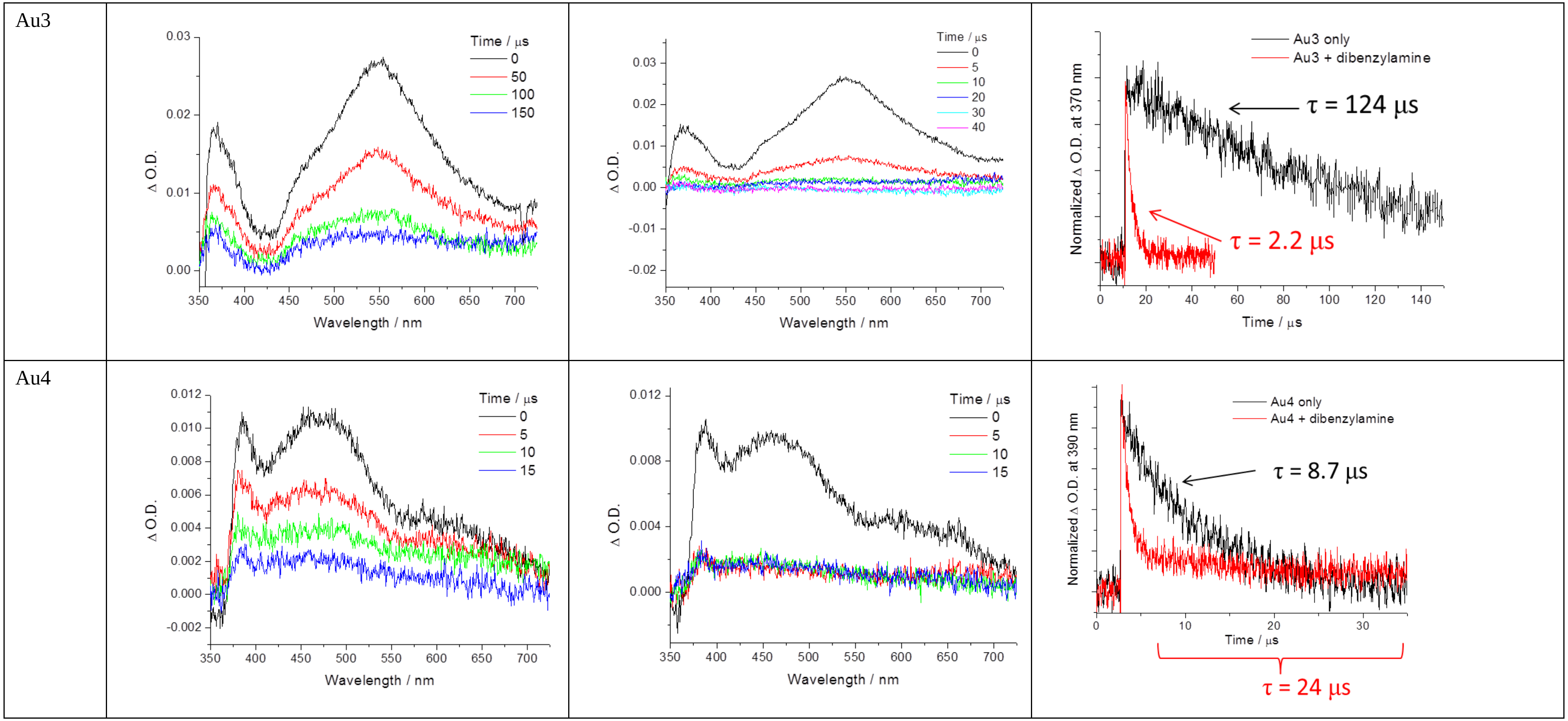

[a] Concentrations: 3×10^{-5} M for Au1 and Au4, 1×10^{-5} M for Au2 and Au3.

The nanosecond time-resolved transient absorption (TA) measurements of solutions of these complexes alone and in the presence of amine substrate in degassed DMF were conducted to examine whether photochemical oxidation of dibenzylamine by excited gold(III) complex is due to electron transfer. For Au1–Au4, all of their TA decay time constants (0.62–124 μs) are close to their respective emission lifetimes, indicating that these absorptions arise from the ^{3}IL excited state of the gold(III) complexes. In the

presence of 0.1 M dibenzylamine, the TA of Au2 and Au3 was quickly quenched (1.4 μs for Au2; 2.2 μs for Au3) and returned to the baseline, suggesting that the observed quenching does not involve electron transfer (W.-P. To, Y. Liu, T.-C. Lau and C.-M. Che, *Chem. Eur. J.*, 2013, **19**, 5654-5664). However, the TA of Au1 and Au4 evolved to give long-lived species with lifetimes of 145 μs and 24 μs, respectively. These long-lived species may be attributed to the formation of reduced gold(III) complexes (i.e. **Au1**$^{-}$ and **Au4**$^{-}$) after the reaction of excited Au1 and Au4 with dibenzylamine (P.-K. Chow, G. Cheng, G. S. M. Tong, W.-P. To, W.-L. Kwong, K.-H. Low, C.-C. Kwok, C. Ma, and C.-M. Che, *Angew. Chem. Int. Ed.*, 2015, **54**, 2084-2089). Thus, the photochemical oxidation of dibenzylamine with Au2 and Au3 is likely to be due to photosensitization of singlet oxygen by excited Au2 and Au3, whereas that with Au1 and Au4 may involve electron transfer in addition to generation of singlet oxygen by excited Au1 and Au4.

Table S8 Nanosecond time-resolved transient absorption (TA) measurements of solutions of Au2 alone and in the presence of *N*-phenyl-1,2,3,4-tetrahydroisoquinoline substrate in degassed DMF.[a]

Complex	Transient absorption spectra of Au2 in degassed DMF	Transient absorption spectra of Au2 with 0.01 M *N*-phenyl-1,2,3,4-tetrahydroisoquinoline in degassed DMF	Decay of transient absorption intensity at specified wavelength
Au2	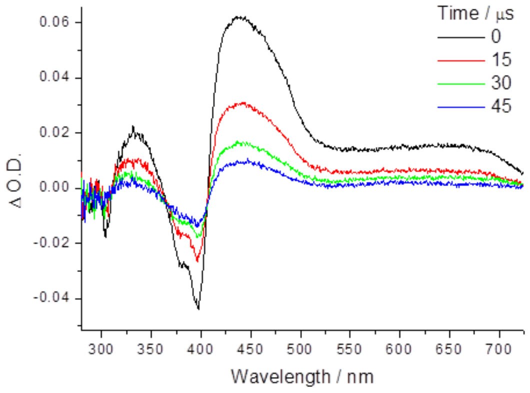	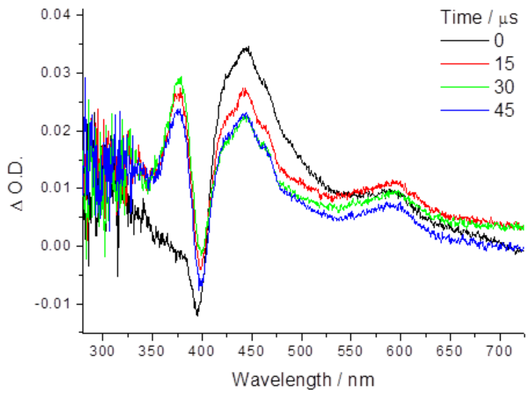	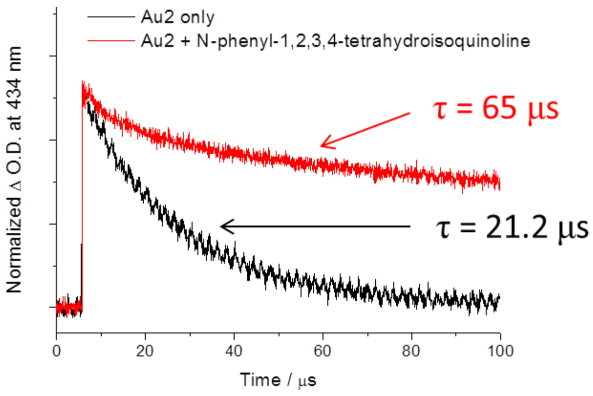

[a] Concentration of Au2: 1×10^{-5} M.

The TA spectrum of Au2 with *N*-phenyl-1,2,3,4-tetrahydroisoquinoline (0.01 M) shows the formation of a long-lived species (τ = 65 μs) which may be attributed to reduced Au2 (i.e. **Au2**$^{-}$). Thus, the photo-induced oxidative functionalization of *N*-phenyl-1,2,3,4-tetrahydroisoquinoline by Au2 may involve both direct electron transfer and photosensitization of singlet oxygen by excited Au2.
